# Differences of the tumour cell glycocalyx affect binding of capsaicin-loaded chitosan nanocapsules

**DOI:** 10.1038/s41598-020-79882-y

**Published:** 2020-12-31

**Authors:** Lydia von Palubitzki, Yuanyuan Wang, Stefan Hoffmann, Sabine Vidal-y-Sy, Bernd Zobiak, Antonio V. Failla, Petra Schmage, Axel John, Anayancy Osorio-Madrazo, Alexander T. Bauer, Stefan W. Schneider, Francisco M. Goycoolea, Christian Gorzelanny

**Affiliations:** 1 Experimental Dermatology, Department of Dermatology and Venereology, University Medical Center Hamburg-Eppendorf, Research Campus, Martinistraße 52, 20246 Hamburg, Germany; 2 Institute of Plant Biology and Biotechnology (IBBP), University of Münster, Schlossplatz 7-8, 48143 Münster, Germany; 3 Microscopy Imaging Facility, University Medical Center Hamburg-Eppendorf, Research Campus, Martinistraße 52, 20246 Hamburg, Germany; 4 Clinic of Periodontology, Preventive and Operative Dentistry, Center of Dental and Oral Medicine, University Medical Center Hamburg-Eppendorf, Martinistraße 52, 20246 Hamburg, Germany; 5 Department of Urology, University Medical Center of Ulm, Albert-Einstein-Allee 23, 89081 Ulm, Germany; 6 Institute of Microsystems Engineering (IMTEK), Freiburg Materials Research Center (FMF), and Freiburg Center for Interactive Materials and Bioinspired Technologies (FIT), University of Freiburg, 79104 Freiburg, Germany; 7 School of Food Science and Nutrition, University of Leeds, Woodhouse Lane, Leeds, LS2 9JT UK

**Keywords:** Biological techniques, Biophysics, Cancer, Cell biology, Oncology, Urology, Nanoscience and technology

## Abstract

The glycocalyx regulates the interaction of mammalian cells with extracellular molecules, such as cytokines. However, it is unknown to which extend the glycocalyx of distinct cancer cells control the binding and uptake of nanoparticles. In the present study, exome sequencing data of cancer patients and analysis of distinct melanoma and bladder cancer cell lines suggested differences in cancer cell-exposed glycocalyx components such as heparan sulphate. Our data indicate that glycocalyx differences affected the binding of cationic chitosan nanocapsules (Chi-NCs). The pronounced glycocalyx of bladder cancer cells enhanced the internalisation of nanoencapsulated capsaicin. Consequently, capsaicin induced apoptosis in the cancer cells, but not in the less glycosylated benign urothelial cells. Moreover, we measured counterion condensation on highly negatively charged heparan sulphate chains. Counterion condensation triggered a cooperative binding of Chi-NCs, characterised by a weak binding rate at low Chi-NC doses and a strongly increased binding rate at high Chi-NC concentrations. Our results indicate that the glycocalyx of tumour cells controls the binding and biological activity of nanoparticles. This has to be considered for the design of tumour cell directed nanocarriers to improve the delivery of cytotoxic drugs. Differential nanoparticle binding may also be useful to discriminate tumour cells from healthy cells.

## Introduction

The glycocalyx is a multifunctional layer of glycans that surrounds all mammalian cells^1,2^. This outer glycan layer is the interface between cells and the extracellular space, and it controls processes such as intracellular signalling, cell adhesion and endocytosis^3^. The glycocalyx comprises highly-glycosylated membrane-associated proteins including proteoglycans such as syndecans and secreted glycosaminoglycan polymers such as hyaluronic acid^1,4^. Proteoglycans carry several covalently attached glycosaminoglycan chains such as heparan or chondroitin sulphate^3,5^. Although glycosaminoglycans are complex molecules with diverse chemical structures, they universally carry a strong negative charge. Previous studies suggest that the high charge density along polyelectrolyte carbohydrate chains such as heparan sulphate is capable of inducing tight counterion binding, which strongly screens the negative charge and is known as counterion condensation^6,7^.

A growing body of evidence indicates that the glycocalyx of cancer cells differs from that of healthy cells because the profile of proteoglycans and glycoproteins is modified^8–11^. Also, there might be a higher degree of glycosylation^1,12^, which appears to be further connected to cancer-related signal transductions^13,14^. In this context, the tumour-promoting effect of the glycocalyx has frequently been linked to its ability to bind growth factors and cytokines^15^. The glycocalyx may also refine the activity of integrins, promoting tumour cell migration and interaction with extracellular matrix components^8,16^.

Advances in transcriptome and exome sequencing showed that tumour tissues are heterogeneous and composed by distinct populations of tumour cells. Melanoma and bladder cancer belong to the most heterogeneous tumour types^17,18^. This heterogeneity affects the therapeutic effects of applied drugs and favours the development of cancer drug resistance^19^. To which extend this cellular heterogeneity is also mirrored by a changed composition of the glycocalyx is unclear. Recently, a glycosaminoglycan-binding protein from the malaria pathogen *Plasmodium falciparum* was used to target distinct glycans of cancer cells, envisioning that knowledge of the cancer cell glycocalyx may improve drug targeted delivery^20,21^. Previous studies suggest that the glycocalyx of mammalian cells plays a key role in the binding and internalisation of nanoparticles^22–24^. However, the impact of the potential heterogeneity of the cancer cell glycocalyx on nanoparticle binding and drug delivery is unknown.

The aim of this work was to characterise the glycocalyx structure and density of human cells that originated from different tissues and differ in their tumour-progressing phenotype. In further experiments, we aimed to correlate the extent of the glycocalyx with its ability to interact with positively charged Chi-NCs. The applied Chi-NCs are composed of an oily, lecithin-covered core and a polycationic chitosan shell^25,26^. Chitosan is a linear polysaccharide consisting of β(1 → 4)-linked units of glucosamine and *N*-acetylglucosamine, with a degree of acetylation usually below 40%, thus appearing as a polycation in aqueous media due to the protonation of the glucosamine amine groups^27–29^. Chitosan is the main derivative of chitin, a natural structural polysaccharide mainly extracted from crustacean shells, cephalopods endoskeleton and fungal cell wall. We considered chitosan as a reasonable model compound, as a large number of in vitro and in vivo studies applied chitosan and chitosan-dependent formulations because of its biodegradability and low cytotoxicity^30–33^. The oily core of Miglyol was loaded with the lipophilic fluorescent dyes 3,3′-dioctadecyloxacarbocyanine perchlorate (DiO), allowing the particles to be tracked and evaluated. We also investigated the ability of capsaicin-loaded Chi-NCs to deliver capsaicin and to induce apoptosis^34^.

## Results and discussion

### Characterisation of the glycocalyx of different human cells

**Figure 1:**
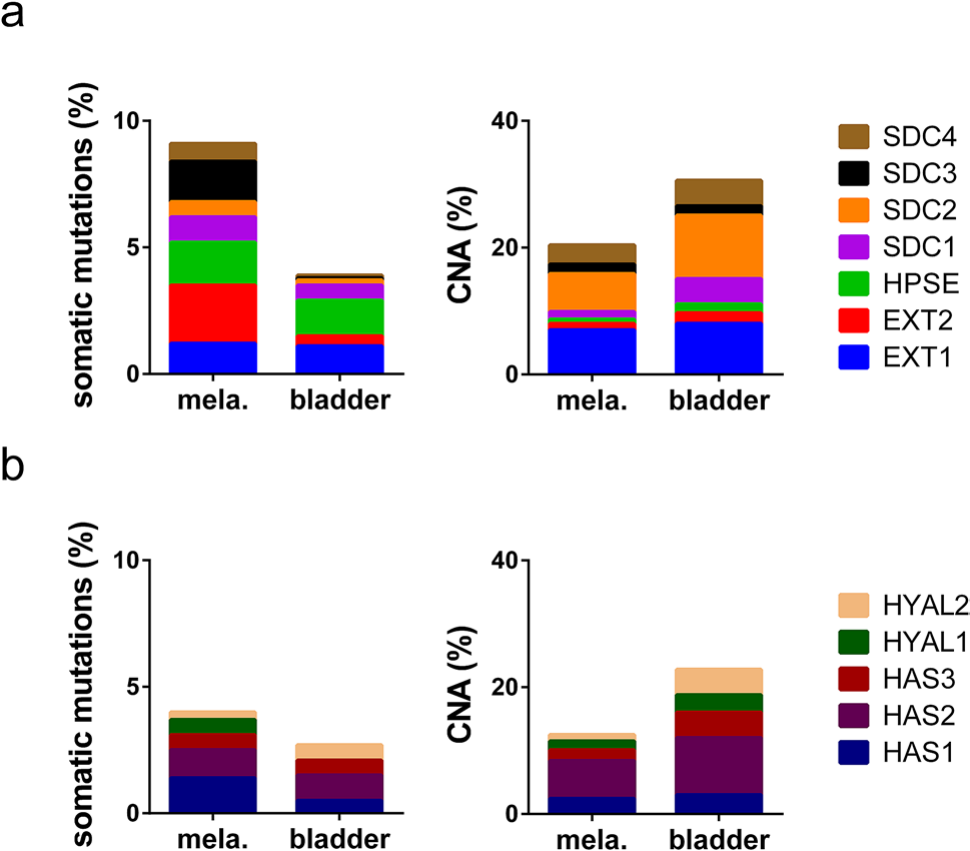
Genetic alterations in glycocalyx-related genes. (**a**,**b**) Screening of public exome sequencing data indicate somatic mutations and copy number alterations (CNAs) in tissues of melanoma (mela.) and bladder cancer (bladder). (**a**) Shown are the percentages of patients with genetic alterations in the proteoglycans syndecan 1 (*SDC1*), *SDC2*, *SDC3* and *SDC4*, the heparan sulphate degrading heparanase (*HPSE*), exostosin glycosyltransferase 1 (*EXT1*) and *EXT2.* (**b**) Shown are the percentages of patients with genetic alterations in hyaluronidase 1 *(HYAL1)* and *HYAL2* and hyaluronan synthases *(HAS1)*, *HAS2* and *HAS3*.

Exploration of public exome sequencing data of bladder cancer and melanoma tissue revealed that genes coding proteoglycans, enzymes of the heparan sulphate or hyaluronic acid biosynthesis and enzymes degrading heparan sulphate or hyaluronic acid are frequently mutated or affected by copy number alterations (CNAs). Figure 1 shows that the frequency of somatic mutations was higher in melanoma than in bladder cancer. In contrast, CNAs were more frequently detected in bladder cancer tissue and less often in melanoma. These data suggest that the glycocalyx of tumour cells of both entities is heterogeneous and varies between different patients and most likely even between individual cells within the same tumour tissue.

**Figure 2:**
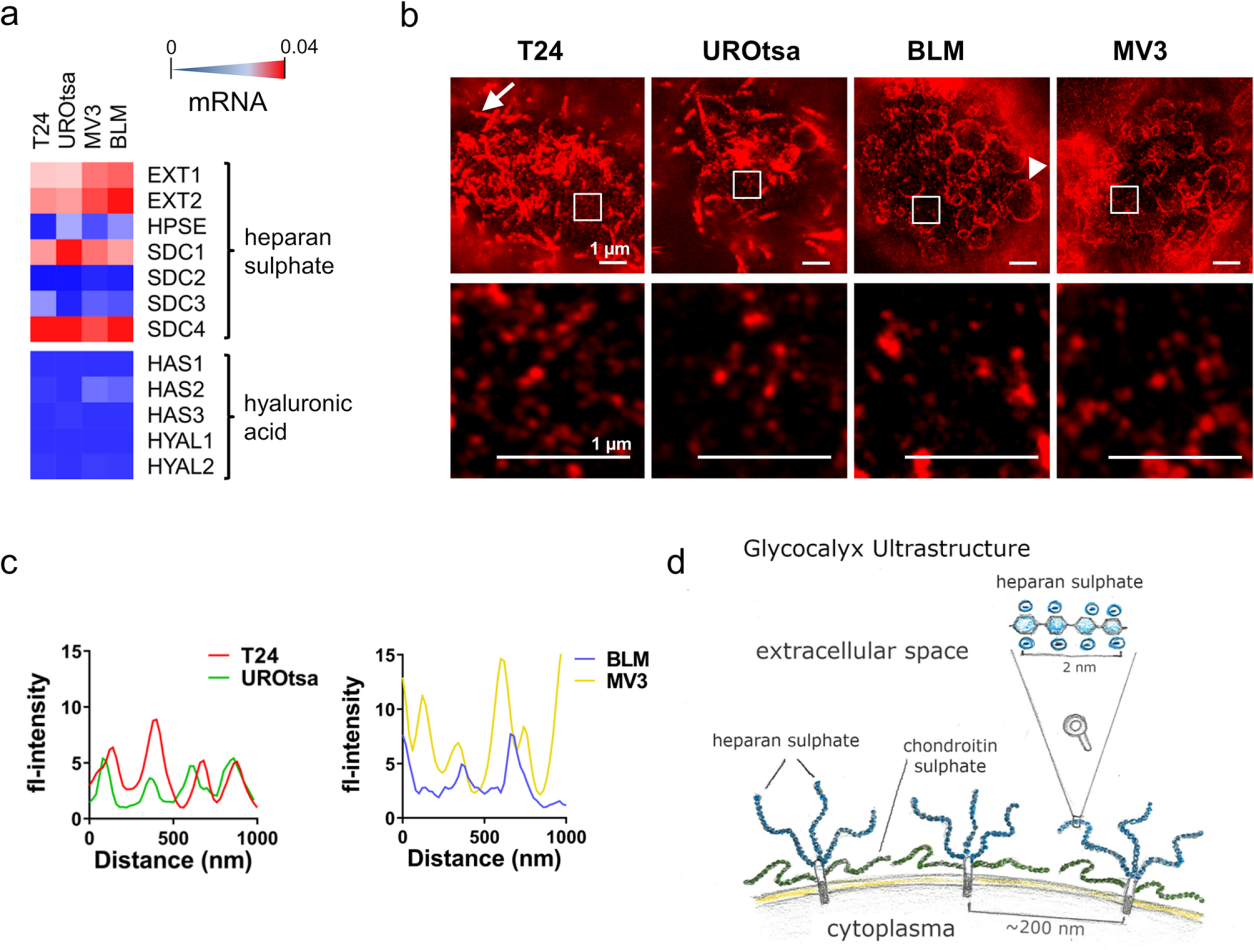
Characterisation of the glycocalyx. (**a**) Analysis of genes involved in heparan sulphate and hyaluronic acid biosynthesis in T24 (bladder cancer cells), UROtsa (benign urothelial cells), BLM and MV3 (melanoma cell lines) by qRT-PCR, with relative expression levels increasing from blue (not expressed) to red (strongly expressed). Measurements were done as multiple PCR runs in triplicates; corresponding data are presented in Supplementary Table S1. (**b**) Stimulated emission depletion microscopy images of the glycocalyx of T24, UROtsa, BLM and MV3 showing that the surface morphology was strongly dependent on the origin of the cell. T24 and UROtsa cells exposed tube-like cellular protrusions (white arrow), whereas BLM and MV3 cells produced bleb-like protrusions (white arrowhead). Scale bars = 1 µm. The shown inserts were placed in dark image areas to prevent overlapping of multiple fluorescence signals but to enable the resolution of single glycan spots. Glycans were stained by ATTO 646 N-conjugated WGA. (**c**) Profile of the stimulated emission depletion microscope images indicating that the average space between the glycoprotein clusters is approximately 200 nm. (**d**) Schematic drawing of cell membrane bound proteoglycans.

Next, we analysed the glycocalyx of urothelial (T24, UROtsa) and melanoma (BLM, MV3) cells of different origin and phenotype. T24 cells are derived from transitional bladder cancer, while UROtsa cells resemble the healthy urothelium^35–37^. The malignant melanoma cell line BLM has been isolated from lung metastasis^38,39^, whereas MV3 cells originated from lymph node metastasis^40^. Figure 2a shows the analysis of glycocalyx-related gene expression by quantitative real time PCR (qRT-PCR). Genes encoding enzymes involved in heparan sulphate synthesis, such as exostosin glycosyltransferase 1 (*EXT1*) and *EXT2*, were strongly expressed, as were those encoding the heparan sulphate proteoglycans syndecan 1 (*SDC1*) and *SDC4*. *SDC2* was weakly expressed by MV3 cells. SDC3 was detected at low levels in T24, and both melanoma cells. In none of the analysed cell lines we detected the expressions of hyaluronan synthase 1 (*HAS1*). *HAS2* was weakly expressed by T24, MV3 and BLM cells. *HAS3* was found in UROtsa cells only. Hyaluronidase 1 (*HYAL1*) expression was below the detection limit in all tested cell lines, while *HYAL2* was weakly expressed by T24, MV3 and BLM cells. (Fig. 2a, Supplementary Table S1). In summary, qRT-PCR data suggest that in all tested cell lines heparan sulphate is more abundant than hyaluronic acid. However, the biosynthesis of glycosaminoglycans is a complex process. For example, heparan sulphate biosynthesis involves the action of 11 different enzymes, which cooperate with and antagonise each other, thus preventing a straight forward correlation between gene expression levels and the quantity of heparan sulphate found on the cell surface^41,42^. Therefore, to further characterise the glycocalyx ultrastructure, we applied stimulated emission depletion microscopy. Cell surface-exposed glycans were stained by ATTO 646N-conjugated wheat germ agglutinin (WGA). We found that the surface morphology was strongly dependent on the origin of the cell (Fig. 2b). While the surface of the urothelial cells (UROtsa and T24) was characterised by tube-like membrane protrusions (Fig. 2b, white arrow), both melanoma cell lines (BLM and MV3) showed bleb-like bulges (Fig. 2b, white arrowhead). The formation of membrane protrusions has been mechanistically linked to the density of the mammalian cell glycocalyx^43,44^. Therefore, tube-like membrane folding, as found on the urothelial cells, may account for a high glycan density. In contrast, bleb-like structures may indicate a low glycan density.

Proteoglycans are organised as clusters on the cell surface^45^ in a tuft-like manner with interstitial gaps in the hundred-nanometer range^46,47^. In line with this concept, we detected single glycoprotein clusters on the surface of all cell lines as small dots (Fig. 2b), with an average spacing of ~ 200 nm (Fig. 2c) and a number of approximately 20 clusters per µm^2^ (T24: 22 ± 2.9 clusters µm^−2^; UROtsa: 21 ± 1.4 clusters µm^−2^; BLM: 18 ± 2.5 clusters µm^−2^; MV3: 18 ± 1.4 clusters µm^−2^)^48^. Figure 2d shows a schematic drawing of transmembranous proteoglycans and the supposed presentation of glycosaminoglycans at the cellular surface.

### Glycocalyx-dependent binding of Chi-NCs

**Figure 3:**
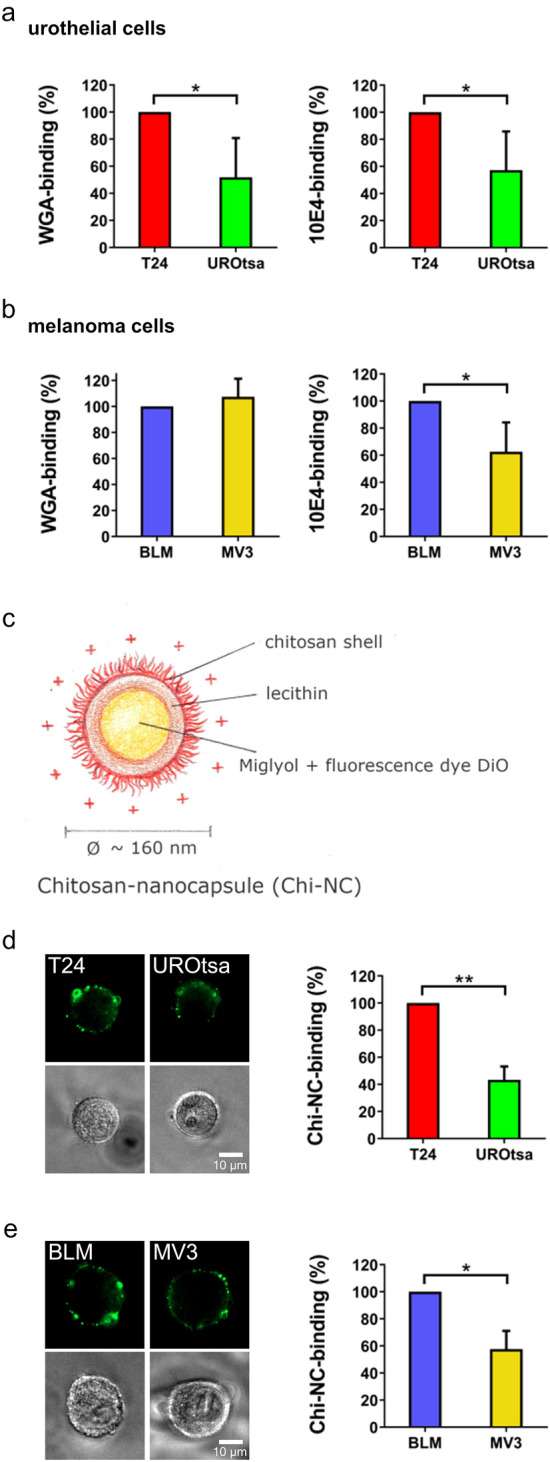
Expression of glycans and Chi-NC binding capacity of urothelial and melanoma cells. (**a**,**b**) Comparison of the WGA-binding ability and heparan sulphate expression (10E4-binding) of urothelial cells (**a**) and melanoma cells (**b**). (**c**) Schematic illustration of Chi-NCs. (**d**,**e**) Urothelial cells (**d**) and melanoma cells (**e**) were treated with Chi-NCs (1.78 × 10^12^ particles mL^−1^). Prior to flow cytometry, binding of Chi-NCs (green) to the cellular surface was verified by fluorescence microscopy (upper panels). Presence of cells was proven by phase-contrast microscopy (lower panel). Scale bars = 10 µm. The bar diagram shows the quantitative evaluation of flow cytometric results. Data were normalised to the corresponding mean fluorescence of T24 or BLM cells. Data are presented as mean ± SD (*n* = 3–4, *p < 0.05, **p < 0.01).

The amount of surface-exposed glycans and in particular of heparan sulphate was determined by flow cytometry (Fig. 3, Supplementary Fig. S1). The applied WGA recognises sialic acids and N-acetylglucosamines, which is a principal component of heparan sulphate, hyaluronic acid and N- and O-linked glycans. For the detection of heparan sulphate only, we used the specific 10E4 monoclonal antibody.

WGA- and 10E4-binding to urothelial cells was of a comparable proportion suggesting that heparan sulphate is a major component of the urothelial glycocalyx and that in average T24 cells expose more heparan sulphate than UROtsa cells (Fig. 3a). Moreover, UROtsa cells showed a less homogeneous heparan sulphate exposure than T24 cells (Supplementary Fig. S1). In melanoma cells (Fig. 3b), we found comparable levels of WGA-binding, whereas the 10E4-binding indicates increased levels of heparan sulphate in BLM cells. The surface expression of glycan structures was further correlated to the ability of the cells to bind Chi-NCs. The Chi-NCs, schematically shown in Fig. 3c, have previously been characterised by our group using dynamic light scattering and transmission electron microscopy^25^. We re-evaluated their size and charge by Zetasizer measurements. They had a diameter of 157 ± 53 nm, a size polydispersity index of 0.2 ± 0.03 and a ζ-potential of + 49.5 ± 8.1 mV. Nanoparticle tracking analysis revealed a comparable diameter of 112 ± 30.2 nm.

Flow cytometry cannot discriminate between surface-bound and intracellular Chi-NCs. Because we aimed to measure the ability of the cells to bind Chi-NCs to their surface, we performed our experiments on ice slowing down Chi-NC endocytosis. To verify the cellular localisation of the Chi-NCs, we analysed the cell suspensions by fluorescence and phase-contrast microscopy prior to flow cytometry. Representative images, shown in Fig. 3d and e, confirmed that the majority of Chi-NCs were in close proximity to the cell membrane. In our quantitative analyses by flow cytometry, we assumed that the cell surface areas of all cells were of an equivalent magnitude. T24 cells bound more Chi-NCs than UROtsa cells which correlated with the elevated amounts of cell surface-exposed glycans (Fig. 3d). Also BLM cells, which expose more heparan sulphate than MV3 cells, bound more Chi-NCs suggesting again the contribution of heparan sulphate in particle binding (Fig. 3e). The discrepant binding of WGA and 10E4 to melanoma cells may indicate the presence of further glycans at the cell surface. Our qRT-PCR results suggest that in comparison to BLM cells, MV3 cells may carry more hyaluronic acid because they express higher levels of *HAS2.* (Fig. 2a).

Taken together, our data indicate that the tumour cell glycocalyx and especially heparan sulphate contribute to the binding of Chi-NCs to the surface of the here tested cancer cells. However, it is likely that also other glycosaminoglycans such as chondroitin sulphate or heavily glycosylated proteins such as mucins could trap cationic nanoparticles at cellular surfaces.

The bladder, and local neoplastic tissues within it, can be accessed by endoscopic surgery, and anti-tumour drugs could be administered via catheters to minimise systemic adverse effects. The instillation of nanoparticles in defined buffers or media may also improve nanoparticle stability and may prevent unintentional modifications such as the formation of protein coronas. The bladder thus appears to be an ideal target for nanotherapeutics. Therefore, we focused on the bladder cancer cell line T24 and the urothelial cell line UROtsa in the further course of our work.

### Internalisation and processing of Chi-NCs

We loaded Chi-NCs with the model lipophilic cytotoxic drug capsaicin, which was previously shown to induce apoptosis in bladder cancer cells^34^. Capsaicin is insoluble in water, which limits its application as a free drug. Accordingly, we found that the addition of free capsaicin to the culture medium of urothelial cells resulted in its precipitation and the generation of crystals (Supplementary Fig. S2). In contrast, capsaicin can be loaded efficiently into the o/w emulsion of Chi-NCs due to its partitioning between the two phases^25^. We have previously shown that the association efficiency of capsaicin was above 90%. The release rate of capsaicin from the Chi-NCs in cell culture medium is low and only in the range of 0.3% per hour^25^.

**Figure 4:**
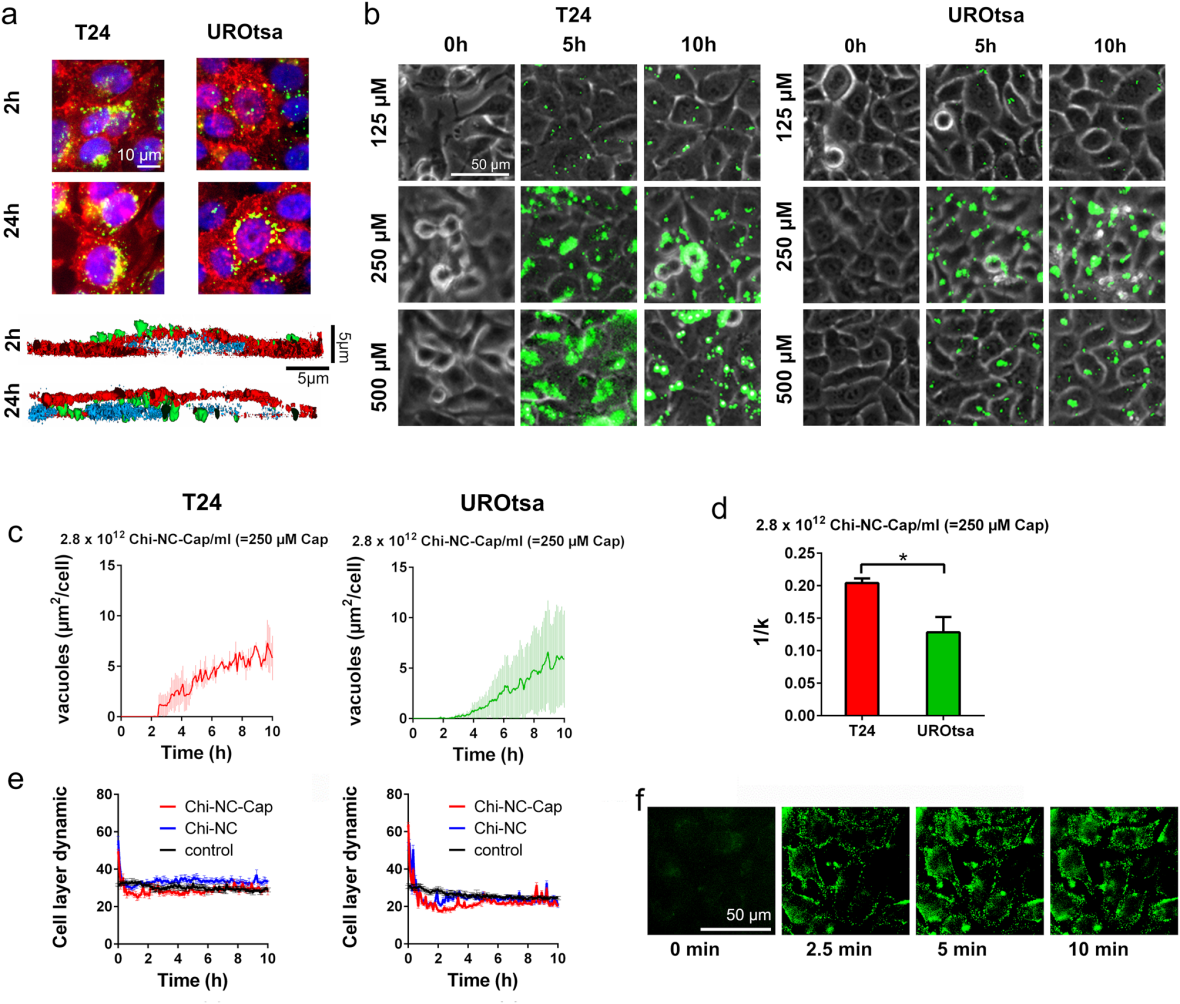
Influence of encapsulated capsaicin on T24 and UROtsa cells. (**a**) Internalisation of fluorescent Chi-NCs (green) into T24 or UROtsa cells was measured by structured illumination microscopy. Nuclei are stained with 4′,6-diamidino-2-phenylindole (blue), cell surfaces (red) were stained with WGA. The upper panel shows two-dimensional images of cells treated for 2 or 24 h with Chi-NCs (2.8 × 10^12^ particles mL^−1^). Scale bar = 50 µm. The lower panel shows a lateral cross-section of UROtsa cells which has been reconstructed from three-dimensional image stacks. Scale bars = 5 µm. After 2 h of incubation, Chi-NCs are mainly at the cellular surface; after 24 h of incubation, Chi-NCs are underneath the cell surface close to the nucleus (**b**) Snapshots of live cell fluorescence microscopy images of UROtsa and T24 cells treated with different concentrations of capsaicin-loaded Chi-NCs (1.4 × 10^12^ particles mL^−1^ = 125 µm capsaicin, 2.8 × 10^12^ particles mL^−1^ = 250 µm capsaicin, and 5.6 × 10^12^ particles mL^−1^ = 500 µm capsaicin) at different points in time (0 h, 5 h and 10 h). Scale bar = 50 µm. (**c**) Quantitative analysis of vacuole formation. Vacuole area is normalized to the number of treated cells. Data are presented as mean ± SD (*n* = 3). (**d**) Quantitative analysis of the ability of the cells to deposit capsaicin in vacuoles (*k*^−*1*^). Data are presented as mean ± SD (*n* = 3, *p < 0.05). (**e**) Analysis of cell layer dynamics of T24 (left) and UROtsa cells (right) either treated with RPMI-1640 medium (control) or treated with capsaicin-loaded Chi-NCs (= Chi-NC-Cap) at a concentration of 500 µm capsaicin (5.6 × 10^12^ particles mL^−1^) and with Chi-NCs without capsaicin (5.6 × 10^12^ particles mL^−1^). Data are presented as mean ± SD (*n* = 3). (**f**) Snapshots of live cell fluorescence microscopy images of T24 cells, 0, 2.5, 5 and 10 min after treatment with DiO-loaded Chi-NCs (5.6 × 10^12^ particles mL^−1^) indicating the rapid binding of Chi-NCs to the cell surfaces. Scale bar = 50 µm.

In the next set of experiments, we applied structured illumination microscopy to measure Chi-NC binding and uptake by adherent T24 and UROtsa cells. Representative images are shown in Fig. 4a. After 2 h of Chi-NC treatment, most of the Chi-NCs are close to the upper surface of the cells. After 24 h of permanent exposure, Chi-NCs appeared to be partially accumulated in perinuclear regions, suggesting Chi-NC uptake via endocytosis followed by endosomal deposition^49,50^. We further investigated the time-dependent Chi-NC internalisation and the potential impact of different capsaicin concentrations (125, 250 and 500 µm capsaicin) on UROtsa and T24 cells by live-cell fluorescence microscopy (Fig. 4b). Representative videos of cells treated with 250 µm capsaicin are shown in Supplementary Video S1 and S2. After 5 and 10 h, cells treated with 250 or 500 µm encapsulated capsaicin formed vacuoles in a dose-dependent manner, indicating cellular stress and autophagy due to the intracellular engulfment of the cytotoxic drug^51,52^. Only few vacuoles were visible in cells treated with 125 µm capsaicin. For quantitative analysis, the formation of vacuoles was measured over time (Fig. 4c, Supplementary Fig. S3) and the data were fitted to Eq. (1):

1 $$N_{v} \left( t \right) = N_{v, max} \left[ {\frac{{t^{n} }}{{\left( {k^{n} + t^{n} } \right)}}} \right]$$

where *N*_*v*_ is the number of vacuoles (experimentally measured in the images as an area fraction), *N*_*v*_*,*_*max*_ is the maximum detectable number of vacuoles, *n* is an arbitrary factor, and *k*^−1^ is proportionally to the ability of cells to deposit capsaicin in vacuoles. Figure 4d shows that vacuoles formed more frequently and faster in T24 cells than in UROtsa cells at a capsaicin concentration of 250 µm. At the highest Chi-NC dose (500 µm capsaicin), T24 and UROtsa cells formed a comparable number of vacuoles (Supplementary Fig. S3). Moreover, after a prolonged incubation time (24 h) and permanent exposure to the capsaicin-loaded Chi-NCs, both cell lines detached from the culture flask. This suggests that the high dose of capsaicin was lethal for T24 but also UROtsa cells and that the per se reduced Chi-NC internalisation by UROtsa cells was overridden.

Because capsaicin can also promote cell migration^53,54^, the dynamic behaviour of the cells was analysed by calculating frame-to-frame differences in our videos. In comparison to untreated cells (control), cellular motilities of T24 and UROtsa cells were slightly higher for few minutes after the administration (time point 0 h) of Chi-NCs and Chi-NC-Cap (5.6 × 10^12^ particles mL^−1^ = 500 µm capsaicin). After this period of increased cellular motility, movement activity of Chi-NC/Chi-NC-Cap treated cells decreases and falls slightly below the control level, until it returned to normal. Of note, changes of the cell layer dynamics appeared to be capsaicin independent because capsaicin-free and capsaicin-loaded Chi-NCs showed a comparable effect. This is also in line with the low release rate of capsaicin from the Chi-NCs suggesting only minor levels of free drug, which are insufficient to induce cell migration (5.6 × 10^12^ particles mL^−1^ = 500 µm capsaicin, Fig. 4e). Live fluorescence microscopy showed that the Chi-NCs bound rapidly to the cells (Fig. 4f) and that Chi-NC binding was also associated with cellular detachment, which may coincide with the initially increased cellular motility (Supplementary Video S3).

In summary, our results suggest that Chi-NCs efficiently deliver capsaicin into urothelial cells and that the cytotoxic response is most likely dependent on intracellular vacuole formation.

### Capsaicin-induced cytotoxicity

**Figure 5:**
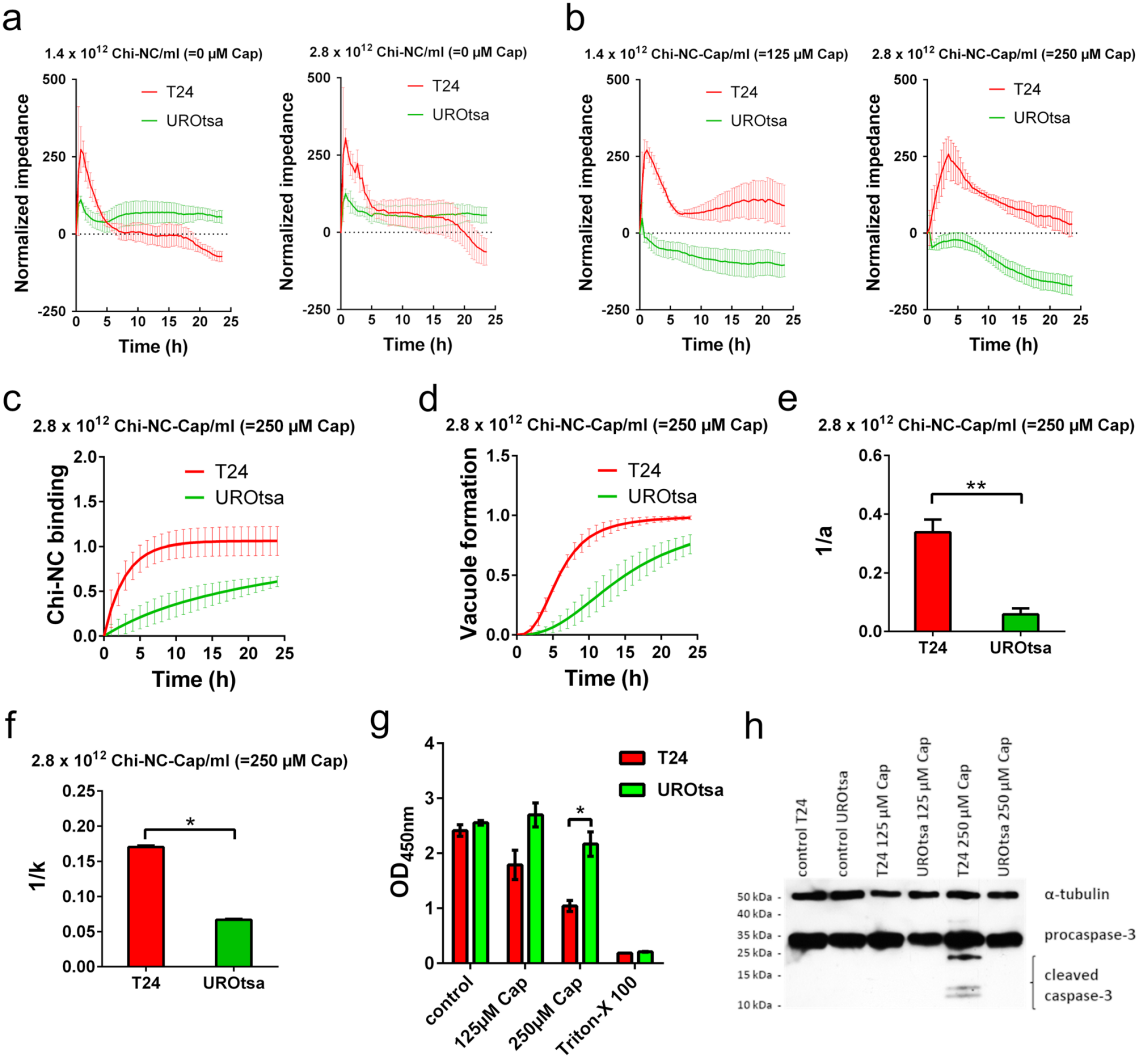
Impedance measurements of T24 and UROtsa cells following treatment with capsaicin-loaded or empty Chi-NCs. (**a**) Control experiments with empty Chi-NCs (no capsaicin) at indicated nanoparticle concentrations (2.8 × 10^12^ and 1.4 × 10^12^ particles mL^−1^). Data are presented as mean ± SD (*n* = 4). (**b**) Treatment with capsaicin-loaded Chi-NCs (Chi-NC-Cap) at indicated concentrations (2.8 × 10^12^ particles mL^−1^ = 250 µm capsaicin, and 1.4 × 10^12^ particles mL^−1^ = 125 µm capsaicin). The response of T24 cells was more pronounced than that of UROtsa cells. Data are presented as mean ± SD (*n* = 4). (**c**) Binding of Chi-NC-Cap as a function of time (2.8 × 10^12^ particles mL^−1^ = 250 µm capsaicin). Data are presented as mean ± SD (*n* = 3). (**d**) Vacuole formation as a function of time upon treatment with Chi-NC-Cap (2.8 × 10^12^ particles mL^−1^ = 250 µm capsaicin). Data are presented as mean ± SD (*n* = 3). (**e**) Derived from (**c**), ability of cells to bind Chi-NC-Cap (*a*^−*1*^). Data are presented as mean ± SD (*n* = 3, **p < 0.01). (**f**) Derived from (**d**), ability of T24 and UROtsa cells to deposit capsaicin in vacuoles (*k*^−*1*^). Data are presented as mean ± SD (*n* = 3, *p < 0.05). (**g**) Viability of Chi-NC-Cap treated T24 and UROtsa cells (2.8 × 10^12^ particles mL^-1^ = 250 µm capsaicin, and 1.4 × 10^12^ particles mL^−1^ = 125 µm capsaicin) was measured by WST-1 assay. RPMI-1640 medium (control) and 1% v/v Triton-X 100 were used as reference. Data are presented as mean ± SD (*n* = 3, *p < 0.05) (**h**) Detection of procaspase-3, cleaved caspase-3 and α-tubulin by western blot in lysates of cells treated with Chi-NC-Cap (1.4 × 10^12^ particles mL^−1^ = 125 µm capsaicin and 2.8 × 10^12^ particles mL^−1^ = 250 µm capsaicin). The unprocessed western blot image is shown in Supplementary Fig. S7.

To confirm the fluorescence microscopy data, we used electrical cell-substrate impedance sensing (ECIS) for real-time quantification of cell behaviour^55^. ECIS or similar methods have frequently been used to measure the loosening of cell–cell contacts^56^. We applied ECIS to measure Chi-NC binding to the cell surface and the formation of vacuoles upon particle endocytosis. To enable a reliable data interpretation, we cultivated T24 and UROtsa cells until they formed a confluent cell layer prior to the treatment with Chi-NCs. Representative microscopic images of cultivated cells are shown in Supplementary Fig. S4a. Absolute impedance of both cell lines was of a comparable magnitude before treatment (Supplementary Fig. S4b). The measured electrical impedance of both urothelial cell lines decreased rapidly after treatment with 500 µm nanoencapsulated capsaicin pointing towards an early cell detachment due to cytotoxicity, preventing reasonable data interpretation (Supplementary Fig. S4c and d). Therefore, we excluded those data from our analysis. After treatment with 125 and 250 µm encapsulated capsaicin or equivalent amounts of empty Chi-NCs (1.4 × 10^12^, 2.8 × 10^12^) particles mL^−1^), we observed an increase in impedance which most likely reflects the binding of Chi-NCs to the cell surface (Fig. 5a,b). This initial increase of impedance peaked after 30 min and then underwent exponential decay. The latter observation may reflect the clearance of Chi-NCs from the cell surface by endocytosis which subsequently resulted in vacuole formation^57^. The response was more pronounced in T24 cells than in UROtsa cells at both Chi-NC concentrations, agreeing with the higher Chi-NC binding and vacuole formation capacity of T24 cells. In principle, ECIS is also sensitive to cell migration which may also account for changes in the measured impedance^58^. However, we consider this effect in our experiment as less relevant, because our microscopic analysis, shown in Fig. 4e, suggested that Chi-NCs affected the migratory potential of urothelial cells only slightly.

We assume that the time-dependent change in the impedance *Z* reflects the number of surface bound Chi-NCs and the formation of vacuoles. Accordingly, we correlated the measured *Z* to the binding of Chi-NCs and to the number of formed vacuoles as shown in Eq. (2):

2 $$Z\left( t \right) = Z_{0} + Z_{max} e^{{ - \frac{t}{a}}} - N_{v}$$

where *Z*_*0*_ is the normalised impedance before Chi-NC treatment (*t* = 0 h), *Z*_*max*_ is the maximum detectable impedance, *a*^−1^ is a factor proportional to the binding of Chi-NCs, and *N*_*v*_ is the number of vacuoles from Eq. (1). Representative fitting of our impedance data is shown in Supplementary Fig. S5.

To facilitate better comparison with our flow cytometry and microscopy data, we segregated the fitted results and plotted the supposed Chi-NC binding and *N*_*v*_ against time (Fig. 5c,d). In addition, Fig. 5e shows factor *a*^−1^ as a measure for Chi-NC binding. The ability of cells to deposit capsaicin in vacuoles (*k*^−1^) is shown in Fig. 5f. T24 cells bound significantly more Chi-NCs than UROtsa cells. Moreover, and in good agreement with our microscopic analysis shown in Fig. 4d, ECIS indicated that T24 cells formed more vacuoles than UROtsa cells (Fig. 5f). This in turn suggests that more capsaicin has been delivered to the T24 cells. No vacuole formation was detected at 125 µm capsaicin because the data fit did not converge to a reasonable result (Supplementary Fig. S6). WST-1 assays confirmed a reduced cell viability of T24 cells which were treated with 250 µm encapsulated capsaicin (Fig. 5g). In comparison, UROtsa cell were only slightly affected. In further experiments, we confirmed the induction of apoptosis by probing western blots of cell lysates for the apoptotic marker cleaved caspase-3 (Fig. 5h and Supplementary Fig. S7). In the presence of 250 µm encapsulated capsaicin, the level of cleaved caspase-3 increased strongly in T24 cells but remained close to the detection level in UROtsa cells. Prolonged exposure of the western blot membrane revealed low levels of cleaved caspase-3 in UROtsa cell treated with 250 µm encapsulated capsaicin (Supplementary Fig. S8). There was no reasonable caspase-3 cleavage in either cell line in response to 125 µm encapsulated capsaicin.

Our data suggest that the enhanced binding of Chi-NCs to T24 cells supports the cytotoxic action of capsaicin, whereas the viability of UROtsa cells was not strongly affected upon Chi-NC binding. We cannot exclude that other cellular mechanisms such as the intracellular processing of capsaicin or the Chi-NCs may account for the measured effects. However, our data illustrate that glycocalyx-dependent differences in nanoparticle binding and internalisation may also affect the biological activities of nanoencapsulated drugs. Our results indicate that T24 cells, in comparison to UROtsa cells, exhibit a pronounced and aberrant glycosylation which increased their susceptibility to nanoparticle-delivered drugs. Previous studies proposed that the ability of cancer cells to metastasize is enhanced by an extended glycocalyx^8^. This implicates that malignant cells were stronger glycosylated than benign cells. However, it appears that this cannot be generalized to every cell line or tumour entity. In melanoma, Cherfils-Vicini et al*.* showed that reduced heparan sulphate levels promote lung metastasis suggesting that malignant cells are less glycosylated^59^. In our own previous work, we showed that elevated heparan sulphate levels on melanoma cells reduce their ability to metastasize into the lung, whereas the formation of lymph node metastasis was promoted^15^. In bladder cancer, an altered expression and composition of glycosaminoglycans such as the oncofoetal chondroitin sulphate has been reported^21^. Taken together these data indicate that the glycocalyx of cancer cells can be altered regarding expression levels and structure. This also implicates an altered capability of cancer cells to interact with nanoparticles compared with benign cells. However whether cancer cells have an increased or decreased capacity to bind nanoparticles might depend on the tumour entity and in particular on tumour cell specific features. Therefore, detailed knowledge of the cancer cell glycocalyx is required to optimize the therapeutic application of nanoencapsulated drugs.

To further characterise the contribution of the glycocalyx to Chi-NC binding and to better understand its molecular basis we analysed the interaction between Chi-NCs and the urothelial cell lines in more detail.

### Cell-specific glycocalyx tunes the electrostatic interaction with Chi-NCs

**Figure 6:**
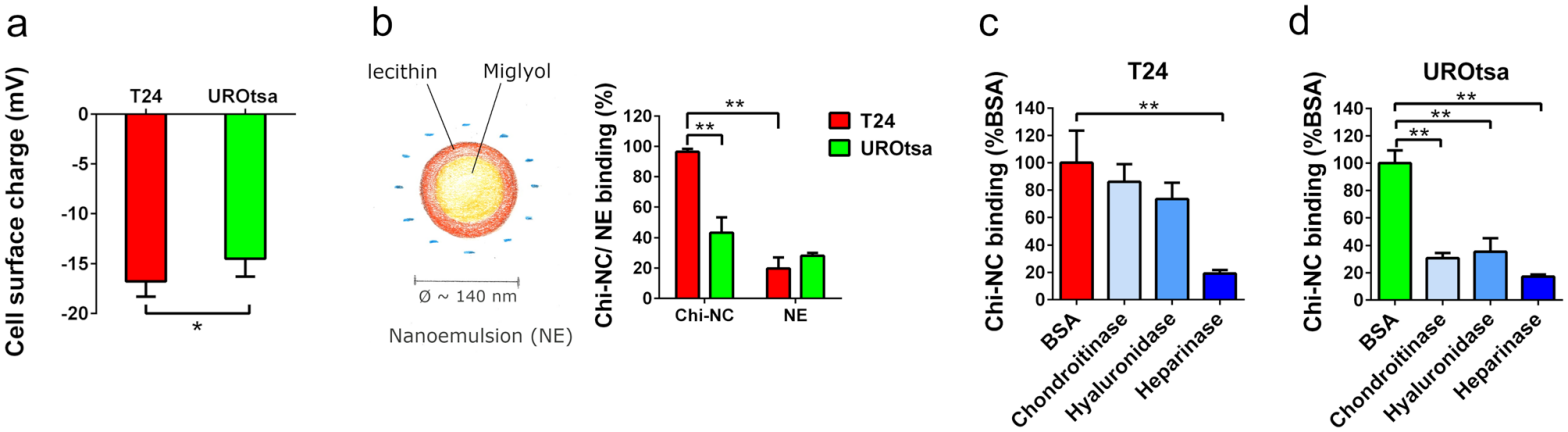
Electrostatic properties of urothelial cells. (**a**) Cell surface charge measurements of T24 and UROtsa cells. Data are presented as mean ± SD (*n* = 3, *p < 0.05). (**b**) Schematic composition of the nanoemulsion (NE) and NE-binding capacity of T24 and UROtsa cells. NE and Chi-NC concentration = 1.78 × 10^12^ particles mL^−1^. Data are presented as mean ± SD (*n* = 3–4, **p < 0.01) and shown in relation to the Chi-NC binding capacity*.* (**c**,**d**) Chi-NC binding of T24 (**c**) and UROtsa cells (**d**) after digestion of the cellular glycocalyx with chondroitinase, hyaluronidase or heparinase. Chi-NC concentration = 8.96 × 10^11^ particles mL^−1^. Bovine serum albumin (BSA) treated cells were used as controls. Data are presented as mean ± SD (*n* = 3, **p < 0.01).

Our data shown in Fig. 3 suggest that heparan sulphate is involved in the Chi-NC binding to urothelial cells. To further underline the expected action of electrostatic forces^60^, we measured the charge density (*ζ*-potential) on urothelial cell surfaces. In line with their higher Chi-NC binding ability, T24 cells are significantly more negatively charged than UROtsa cells (Fig. 6a). Moreover, reduced cellular binding of negatively charged nanoemulsions (diameter = 135 ± 28 nm, *ζ*-potential = − 31.4 ± 4.83 mV, Fig. 6b^25,26^) further confirmed that the interaction between Chi-NCs and the urothelial cells is driven by electrostatic forces (Fig. 6b). Electrostatic interactions have often been exploited for the intracellular delivery of drugs and nucleic acids through different polycationic nanocarrier systems^61–63^.

Seiler and colleges^21^ showed that chondroitin sulphate is a valid therapeutic target on bladder cancer cells. Because chondroitin sulphate is, similar to heparan sulphate, highly negatively charged, it may also contribute to the binding of Chi-NCs to bladder cancer cells. Therefore, to further clarify the contribution of glycocalyx components such as heparan sulphate, chondroitin sulphate and hyaluronic acid, we treated T24 and UROtsa cells with heparinase, chondroitinase and hyaluronidase (Fig. 6c,d). In case of T24 cells, hyaluronidase and chondroitinase treatment had little effect on Chi-NC binding, whereas elimination of heparan sulphate by heparinase prevented Chi-NC binding efficiently. Interestingly, Chi-NC binding to UROtsa cells appeared to be more complex and dependent on hyaluronic acid, heparan and chondroitin sulphate. However, it is worth mentioning that the applied chondroitinase may also exhibit slight activity on hyaluronic acid^64^. UROtsa cells express also more heparanase (HPSE) than T24 cells (Fig. 2a). Accordingly, the intrinsic HPSE of UROtsa cells can partially remove heparan sulphate from the cellular surface also during chondroitinase or hyaluronidase treatment.

### Heparan sulphate related counterion condensation affects Chi-NC binding

It is known from polymer science that the mobility of counterions, and thus their charge screening effect, depends on the charge density of the proximate polyelectrolyte. Previous data suggest a strong charge screening effect along heparan sulphate chains through counterion condensation^6^. Whereas high counterion mobility and moderate charge screening occurs in polyelectrolytes with low charge densities, the counterion mobility approaches zero (counterion condensation) for highly-charged polyelectrolytes, resulting in strong charge screening. We assume that counterion condensation may also influence the electrostatic interaction between oppositely charged mammalian cells and nanoparticles. Counterion condensation may even create a counterintuitive effect, in which cells with high levels of heparan sulphate show weak nanoparticle binding. In contrast, cells with less heparan sulphate may have an increased ability to interact with nanoparticles. The counterion condensation theory^7^ postulates that the dimensionless line charge density *ξ* is a decisive parameter for counterion condensation.

The value of *ξ* is defined as shown in Eq. (3):

3 $$\upxi = \frac{{e^{2} }}{{4\pi \varepsilon_{0} \varepsilon kTb}}$$

where *b* is the average distance between charges along the polyelectrolyte chain, *e* is the elementary charge, $\varepsilon_{0}$ is the vacuum permittivity, $\varepsilon$ is the dielectric constant of the solvent (78.5 for water at 25 °C), *k* is the Boltzmann’s constant, and *T* is the absolute temperature. The value of *b* is calculated as shown in Eq. (4):

4 $$b = \frac{L}{P}$$

where *L* is the contour length of the polyelectrolyte and *P* is the number of charged groups.

The counterion condensation theory predicts that condensation occurs when *ξ* > 1. Heparan sulphate can carry up to four charges per nanometre, which based on Eqs. (3) and (4) gives *b* = 0.25 nm and *ξ* = 2.9, supporting the occurrence of counterion condensation. Comparable electrostatic properties could be supposed for chondroitin sulphate. Hyaluronic acid is less negatively charged and most likely not affected by counterion condensation^65^.

**Figure 7:**
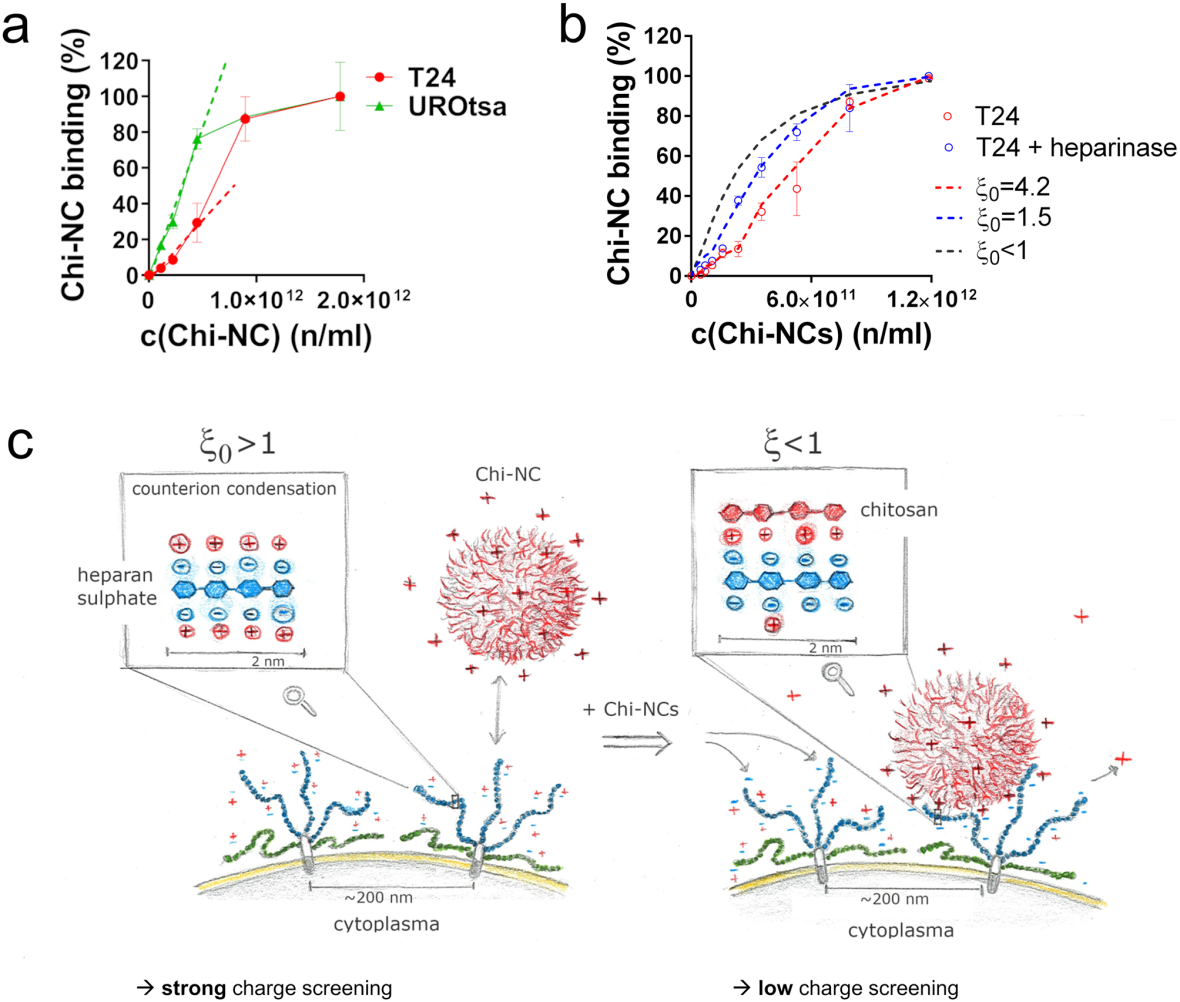
Dose-dependent binding of fluorescent Chi-NCs to T24 and UROtsa cells. (**a**) Chi-NC binding of T24 and UROtsa cells at increasing Chi-NC concentrations (1.12 × 10^11^ to 1.78 × 10^12^ particles mL^−1^) measured by flow cytometry. Data are presented as mean ± SD (*n* = 4). Because UROtsa cells bound significantly less Chi-NCs than T24 cells (Fig. 3d), we normalised the maximum of detectable fluorescence of both cells to 100%. Dashes lines indicate the initial dose-dependent increment of the measured fluorescence. (**b**) Chi-NC binding to control (red circles) or heparinase-treated (blue circles) T24 cells. The grey dashed line indicates in silico simulations of Chi-NC binding to cell surfaces in the absence of counterion condensation (initial charge density (*ξ*_*0*_) < 1), the blue dashed line shows simulation of Chi-NC binding at *ξ*_*0*_ = 1.5 suggesting weak counterion condensation and the red dashed line shows simulation of Chi-NC binding at *ξ*_*0*_ = 4.2, representing strong counterion condensation. Chi-NC concentrations = 4.97 × 10^10^ to 1.27 × 10^12^ particles mL^−1^). (**c**) Schematic illustration of the proposed counterion condensation effect regulating Chi-NC binding to the negatively charged heparan sulphate chains. Prior to the treatment with Chi-NCs, counterions are tightly bound to the heparan sulphate chains due to counterion condensation (left). The initial charge density (*ξ*_*0*_ > 1) results in the strong screening of negative charges by counterions, which lowers the effective charge. Cooperative binding of Chi-NCs to heparan sulphate neutralises some of the negative charges leading to a reduced charge density (*ξ* < 1), no counterion condensation and thus to an increased effective charge that promotes further Chi-NCs binding (right).

In our preceding experiments, we applied high saturating Chi-NC concentrations. Therefore, we expect that the Chi-NC binding was mainly controlled by the total number of available binding sites at the cell surface (Fig. 3). In addition, high concentrations of Chi-NCs ensured also that sufficient capsaicin was delivered into the cells to induce apoptosis (Fig. 5). To analyse the potential impact of charge screening on the binding of Chi-NCs to mammalian cells, we treated both urothelial cell lines with different and lower concentrations of Chi-NCs (1.12 × 10^11^ to 1.78 × 10^12^ particles mL^−1^). Figure 7a shows the dose-dependent Chi-NC binding to UROtsa and T24 cells. To prevent misinterpretations due to the differences in Chi-NC binding capacity of T24 and UROtsa cells (Fig. 3d), we normalised the maximal measured mean fluorescence of T24 and also of UROtsa cells to 100%. At low Chi-NC concentrations and thus below saturation and independent of other limiting factors, such as differences in cell size, particle binding increases linearly with increasing amounts of Chi-NCs. However, the dose-dependent increment of particle binding was 2.5 times steeper in the UROtsa cell group than in the T24 cell group (UROtsa: 17.0 × 10^–11^ (%(Chi-NC binding) ml n^−1^); T24: 6.6 × 10^–11^ (%(Chi-NC binding) ml n^−1^)). Moreover, the binding curve of the T24 cells suggests a cooperative binding behaviour, with a weak binding rate at low Chi-NC doses and an increasing rate towards higher Chi-NC concentrations before saturation is reached. Following the idea of counterion condensation, the weak interaction between T24 cells and Chi-NCs at low doses may reflect the screening of negative charges on the heparan sulphate chains by counterions, which results in less potential binding sites for Chi-NCs.

Recently, counterion condensation on heparin was shown to increase with the heparin chain length, also suggesting an increase in counterion condensation on cells with high levels of heparan sulphate, such as T24 cells^6^. Interestingly and in contrast to T24 cells, Fig. 6b indicated that Chi-NC binding to UROtsa cells was at least partially dependent on hyaluronic acid which is not sufficiently charged to provoke counterion condensation^65^. This may also explain the non-cooperative binding of Chi-NCs to UROtsa cells (Fig. 7a). In further experiments, we analysed Chi-NC binding in a salt-free sucrose buffer, anticipating the promotion of electrostatic interactions and the abolishment of the counterion condensation effect (Figure S9). Indeed, under these conditions, the cooperative binding of Chi-NCs to T24 cells was attenuated indicating less counterion condensation. Because the absence of salt is stressful for mammalian cells, we aimed to confirm the impact of the supposed counterion condensation by additional experiments. Accordingly, we repeated our flow cytometry experiments with T24 cells treated with heparinase. We assumed that a thinner glycocalyx with shorter heparan sulphate chains exhibit less counterion condensation and in turn an improved Chi-NC binding at low particle doses. Although the cooperativity of the cell-particle interaction was not completely abolished, normalised particle binding rate to T24 cells with chopped heparan sulphate chains was increased in comparison to control T24 cells (Fig. 7b). Further in silico modelling of our data enabled us to estimate the initial effective charge, occurring at the cellular surface prior to the addition of Chi-NCs (Fig. 7b, dashed lines). Our model assumed a reduced number of binding sites for Chi-NCs below a critical nanoparticle concentration due to counterion condensation. The related decreased probability of binding events attenuated the number of cell-bound particles. Above the critical Chi-NC concentration, the release of tightly bound counterions increased the number of binding sites and thus the probability of binding events. We further translated the assumed initial effective charge to a corresponding initial charge density (*ξ*_0_) as previously reported^6^. Accordingly, the calculated *ξ*_0_ value of the control T24 was 4.2, thus indicating strong counterion condensation, whereas upon heparinase-treatment *ξ*_*0*_ was assumed to be 1.5, suggesting only weak condensation. Moreover, our data enabled us to determine the critical amount of Chi-NCs, c*, required for the transition from the condensation regime at low particle concentrations to non-compromised counterion mobility at higher Chi-NC doses (Fig. 7b). For transition in the control T24 cells, 2.6 × 10^11^ Chi-NCs ml^−1^ were required; whereas in T24 cells that underwent heparan sulphate degradation the transition occurred already at c* = 1.2 × 10^11^ Chi-NCs ml^−1^. In summary, we postulate that the interaction between mammalian cells with an extended glycocalyx and cationic nanoparticles is diminished at low doses (Fig. 7c, left), while disruption of the counterion condensation regime above a critical amount of nanoparticles releases screened binding sites at the cellular surface which promotes further nanoparticle binding (Fig. 7c, right). By determine c*, we were able to calculate a length of 230 ± 36 or 109 ± 16 nm for the heparan sulphate chains covering non-treated or heparinase-treated T24 cells, respectively (Supplementary Information). This agrees with the previously measured glycocalyx thickness of mammalian cells^8,59^.

The theory of counterion condensation is applicable to several polyions but may not completely describe the effects we measured at the cellular surface^66^. Detailed molecular dynamics simulations may offer an interesting way to understand better the occurrence and relevance of counterion condensation at the glycocalyx of mammalian cells^67^.

## Conclusion

In the past, the glycocalyx of mammalian cells has been investigated extensively to determine its role in cell migration and the perception of extracellular signals, but knowledge on its role for the interaction of cells with nanoparticles is scarce. In line with the public databases, our results suggest that the glycocalyx of different tumour cells of distinct entities are highly heterogeneous, which appear to be further translated to different nanoparticle binding capacities. However, we found that particle binding and the composition as well as the extent of the glycocalyx are not related in a simple manner. The contribution of different glycocalyx components and charge screening effects through counterion condensation must be taken into account. This work focused on the urothelial glycocalyx because the bladder is a hollow organ amenable for direct and local administration of nanoparticles, thus circumventing the limitations of systemic delivery such as the formation of a protein corona and nanoparticle clearance. However, the crosstalk between the glycocalyx and nanoparticles appears to be a common feature affecting the interactions between nanoparticles and all cancer cells. Here, we present a quantitative approach that enables to estimate the critical number of nanoparticles needed to maximise their binding to the glycocalyx of urothelial cells, thus promoting a localised drug delivery. This knowledge may be realised in future applications aiming to the rational dosage of nanoparticles for therapy or imaging purposes. Advanced knowledge of the acting electrostatic forces appears to be required to improve nanoparticle-mediated drug targeting and to prevent the generation of drug resistances during therapy.

## Methods

Additional methods are described in the supplemental methods section (Supplementary Information).

### Cell culture

The human bladder cancer cell line T24 (derived from urinary bladder carcinoma^68,69^), the benign immortalised urothelial UROtsa cell line (kindly provided by Dr. Phillip Erben, Heidelberg University; derived from the urothelium lining the ureter of a 12-year-old girl^35–37,70^) as well as the human metastatic melanoma cell lines MV3 (developed from lymph node metastases^40^), and BLM (subline of BRO, derived from a primary human melanoma, isolated from lung metastasis after subcutaneous inoculation of nude mice with BRO cells^38,39^) were cultured in RPMI-1640 medium (Sigma-Aldrich, Darmstadt, Germany) supplemented with 10% fetal bovine serum (FBS), 1% l-glutamine (Biochrom, Berlin, Germany) and 1% penicillin/streptomycin (Biochrom) as described previously^71^. The cells were incubated in a humid atmosphere at 37 °C with a 5% CO_2_ atmosphere until they achieved a confluency of 90%.

### Preparation and characterisation of nanoformulations

Chi-NCs were prepared according to the solvent displacement technique^61^ with modifications^26^. Briefly, an organic phase was formed by dissolving 40 mg of lecithin (Cargill, Krefeld, Germany) in 1 mL of ethanol, followed by the addition of 530 µL capsaicin solution (Sigma-Aldrich; 96% pure dissolved in ethanol to 24 mg mL^−1^), 125 μL Miglyol 812 (Peter Cremer Oleo, Hamburg, Germany), and the lipophilic dye DiO (Santa Cruz Biotechnology, Heidelberg, Germany) to obtain capsaicin concentration of 10 mM and dye concentrations of 0.158 mM, in the final formulation after evaporation^25^. Unloaded Chi-NCs were prepared by replacing the capsaicin solution with ethanol. The organic phase was made up to 10 mL with ethanol and then immediately poured over 20 mL of an aqueous chitosan solution (0.5 mg mL^−1^ dissolved in water with a 5% stoichiometric excess of 1 M HCl) containing chitosan HMC 70/5 with a degree of acetylation of 32% (as determined by ^1^H NMR) and a molecular weight of 5.01 × 10^4^ g mol^−1^ (Heppe Medical Chitosan, Halle, Germany), the same material as in our previous studies^26^. The Chi-NCs formed spontaneously due to solvent displacement and Marangoni effects^72^. Finally, the ethanol and part of the water were evaporated at 40 °C under vacuum in a R210 Rotavapor (Büchi Labortechnik, Essen, Germany) and the volume of the formulations was reduced to 6 mL^26^. The nanoemulsions were prepared using the same procedure but without including chitosan.

A Zetasizer NanoZS (Malvern Instruments, Malvern, UK) fitted with a red laser (λ = 632.8 nm) was used to measure the size and ζ-potential of the nanoformulations^25^. Diameter of the Chi-NCs was verified by nanoparticle tracking analysis (NanoSight LM10, NanoSight, UK). The concentration of free (non-cell bound) fluorescent Chi-NCs was determined by tracking the DiO-loaded nanoparticles on an Axiovert 200 fluorescence microscope (Zeiss, Oberkochen, Germany) equipped with a 40 × oil objective with a numerical aperture of 1.4 (Zeiss) as previously described^73^. Images of 256 × 256 pixels were recorded using a water-cooled iXon3 camera (Andor Technology, Belfast, UK) at a rate of 10 frames per second and an EMCCD gain of 300. Number of cell bound Chi-NCs was calculated by subtracting the number of free Chi-NCs from the total number of Chi-NCs.

### Stimulated emission depletion microscopy

Stimulated emission depletion microscopy and confocal microscopy were carried out in sequential line scanning mode using an Abberior stimulated emission depletion expert line microscope as previously reported^74^. Briefly, cells were seeded on glass coverslips and cultivated until they reached confluency. After fixation of the cells with 2% paraformaldehyde (Electron Microscopy Sciences) in HEPES buffered Ringer's solution, cell surface glycans were stained with WGA (Sigma-Aldrich) conjugated with an ATTO 646 N protein labelling kit (JenaBioscience, Jena, Germany). The stimulated emission depletion microscopy setup was based on a Nikon Ti-E microscope body for the excitation and detection of the fluorescence signal through a 60 × (numerical aperture 1.4) P-Apo oil immersion objective. A pulsed laser was used for excitation at 640 nm and a near-infrared pulsed laser (775 nm) for depletion. The detected signal was directed through a variable-sized pinhole (set to match 1 Airy at 640 nm) and detected using novel state-of-the-art avalanche photodiode APDs with appropriate filter settings for Cy5 (615–755 nm). Images were recorded with a dwell time of 3 µs, and the voxel size was set to 20 × 20 × 150 nm. Images were acquired in time-gating mode with a gating width of 8 ns and a delay of 781 ps. Glycan clusters on the cell surface were analysed by imageJ software and the density of clusters were calculated similar as previously published^48^.

### Flow cytometry

Cells were collected and incubated with Alexa Fluor 488-conjugated WGA (Thermo Fisher Scientific, Waltham, USA, 1:1000) or with the against heparan sulphate directed antibody (10E4 epitope, AMS Biotechnology, Frankfurt, Germany; 1:400) for 30 min on ice. After washing with phosphate buffered saline (PBS), heparan sulphate antibodies were labelled by Alexa Fluor 488 goat anti-mouse IgM secondary antibodies (Thermo Fischer Scientific); 1:1000) for 30 min on ice. PBS washed cells were analysed by a BD FACSCanto flow cytometer (Biosciences, Erembodegem, Belgium). For Chi-NC binding experiments cells were collected and distributed into tubes (400,000 cells per tube in 600 µL PBS) and Chi-NCs were added at different concentrations. The cells were exposed to 1.78 × 10^12^ Chi-NCs mL^−1^ in the experiments shown in Fig. 3d,e, 6b and Supplementary Fig. S1, to 8.96 × 10^11^ Chi-NCs mL^−1^ in the experiments shown in Fig. 6c,d, to 4.97 × 10^10^, 7.46 × 10^10^, 1.12 × 10^11^, 1.68 × 10^11^, 2.52 × 10^11^, 3.78 × 10^11^, 5.66 × 10^11^, 8.48 × 10^11^, 1.27 × 10^12^ Chi-NCs mL^−1^ shown in the experiments in Fig. 7b and to 1.12 × 10^11^, 2.24 × 10^11^, 4.48 × 10^11^, 8.96 × 10^11^, 1.78 × 10^12^ Chi-NCs mL^−1^ in the experiments shown in Fig. 7a and S9. The mixtures were incubated for 30 min on ice and shaken approximately five times during incubation. At the end of the incubation period, the mixtures were centrifuged at 260×*g* for 5 min, before removing the supernatant and resuspending the cells in 500 µL PBS. Chi-NC binding was studied in tubes containing ~ 200,000 cells by detecting the fluorescent dye DiO with the corresponding detector of a BD FACSCanto flow cytometer (Biosciences). Prior to flow cytometry, Chi-NC binding to the cell surface was verified by fluorescence microscopy.

### Flow cytometry after enzyme treatment

T24 cells were detached and treated in the incubator at 37 °C with 500 mU mL^−1^ heparinase I and II (Sigma-Aldrich) in 0.1% bovine serum albumin (BSA), 150 mg mL^−1^ hyaluronidase (Sigma-Aldrich) in 0.1% BSA buffer, 500 mU mL^−1^ chrondroitinase ABC (AMS Biotechnology) in 0.1% BSA or only in 0.1% BSA as a control for 3 h. The cells were then centrifuged at 260×*g* for 5 min and washed with PBS. The cells were exposed to 8.96 × 10^11^ Chi-NCs mL^−1^.

### ECIS

The impedance of UROtsa and T24 cells was measured as previously described^75^, using an ECIS 16-well station and 8W10E + PET eight-well ECIS slides (both from Applied Biophysics, New York, USA). The ECIS slides were coated with 200 µL gelatin (0.5% w/v) for 30 min and stabilised with 200 µL equilibrated RPMI-1640 medium. UROtsa and T24 cells were seeded into the eight-well plate at a density of 150,000 cells/well in 600 µL RPMI-1640 medium (without FCS but containing 1% l-glutamine and 1% penicillin/streptomycin). The impedance was measured continuously at different frequencies (500–64,000 Hz) for 95 h. After 40 h, the RPMI-1604 medium was refreshed. After 72 h, the Chi-NC treatment solution (dissolved in 100 µL RPMI-1640 medium) was added to the wells, directly into the medium without stopping the measurement or removing the slides from the incubator. Both cell lines were treated with three concentrations of encapsulated capsaicin (125, 250, 500 µm) or equivalent quantities of empty Chi-NCs without capsaicin (1.4 × 10^12^, 2.8 × 10^12^, 5.6 × 10^12^ particles mL^−1^) for control experiments. The impedance measurement was continued for 24 h.

### Cell surface charge measurements

The *ζ*-potential of the UROtsa and T24 cells was measured as previously described^26^. Cells were cultivated until they reached 90% confluency, detached with trypsin and resuspended at a concentration of 100,000 cells mL^−1^ in isotonic, salt-free 30 mM HEPES buffer supplemented with 300 mM sucrose (pH 7.4) and containing different volume fractions of Chi-NCs. The *ζ*-potential was measured by mixed laser Doppler velocimetry and phase analysis light scattering (M3-PALS). Particle size and derived count rate were determined by dynamic light scattering with non-invasive back scattering (DLS-NIBS) at a measurement angle of 173°. The autocorrelation functions were matched to the default non-negative least squares (NNLS) fit to calculate the intensity size distribution plots and thus evaluate the Z-average diameter. A Malvern Zetasizer NanoZS fitted with a red laser (λ = 632.8 nm) was used for both methods and Zetasizer v7.12 was used to acquire and evaluate the data.

### Structured illumination microscopy of cells treated with fluorescent Chi-NCs

UROtsa cells or T24 cells were seeded on glass coverslips until they formed a confluent layer. Cells were treated with DiO-loaded Chi-NCs (2.8 × 10^12^ particles mL^−1^), in RPMI-1640 without FCS for 2 or 24 h. After two washing steps with HEPES buffered Ringer’s solution, the cells were fixed with 4% paraformaldehyde (Electron Microscopy Sciences). Cell surfaces were stained with Texas-red-conjugated WGA (Thermo Fisher) diluted in HEPES buffered Ringer’s solution (1:1000); nuclei were stained with 4′,6-diamidino-2-phenylindole. After three further washing steps, coverslips were embedded into a 1,4-Diazabicyclo-(2,2,2)octan/Mowiol (Sigma Aldrich) and mounted on a glass object slide. Samples were imaged using an Observer z1 inverted microscope equipped with a structured illumination module (ApoTome, Zeiss). Images were analysed with Zen software (version 1.1.2.0, Zeiss).

### Live cell microscopy

T24 and UROtsa cells (150,000 cells/well) were seeded into a chambered microscope slide with eight wells (Ibidi, Gräfelfing, Germany) and maintained at 37 °C in a humid, 5% CO_2_ atmosphere. Cells were imaged by time-lapse phase-contrast and fluorescence microscopy using an Observer z1 inverted microscope at 37 °C and 5% CO_2_ in a humidified incubation chamber. Cells were treated with different concentrations of nanoencapsulated capsaicin (125 µm capsaicin = 1.4 × 10^12^ particles mL^−1^, 250 µm capsaicin = 2.8 × 10^12^ particles mL^−1^, 500 µm capsaicin = 5.6 × 10^12^ particles mL^−1^) or the equivalent quantities of Chi-NCs without capsaicin. Images were captured every 5 min for 10 h (Fig. 4b-e and Supplementary Video S1 and S2) or every 10 s for 10 min (Fig. 4f and Supplementary Video S3). ImageJ v1.52 was used to quantify the dynamics of the cell layer. The applied imageJ macro for automatized image analysis was self-made and can be obtained from the authors upon request. Briefly, phase-contrast images (8-bit format) were converted into a binary image that shows bright regions above a critical threshold in black and regions below a critical threshold value in white. Threshold values were set automatically using the “mean dark” threshold setting. From consecutive binary images, standard deviation was calculated using the imageJ “Z project”. Standard deviation images of one experiment were stacked together and mean brightness of every image was calculated. Change of the mean brightness was considered to be proportional with the migratory potential of cells within the cell layer. To analyse the formation of vacuoles, a similar approach was used (Fig. 4c and Figure Supplementary S3). Briefly, consecutive fluorescence images were loaded into imageJ and areas with specific fluorescence intensity range were defined using the auto threshold setting “triangle dark”. The area of vacuoles was determined using the measurement function of imageJ. Data were further normalised to the average number of cells per field of view.

### Western blot

T24 and UROtsa cells were cultured for 72 h in 24-well plates and then treated with 125 or 250 µm encapsulated capsaicin (1.4 × 10^12^ or 2.8 × 10^12^ particles mL^−1^) for 24 h before western blot analysis^76^. Briefly, proteins were separated in a gradient (4–15%) polyacrylamide gel and transferred to a Transblot Turbo nitrocellulose membrane (both from Bio-Rad, München, Germany). Antibodies against pro-caspase-3 (dilution 1:400) and cleaved caspase-3 (dilution 1:100) were obtained from Cell Signaling Technology (Danvers, USA). Horseradish peroxidase-conjugated goat-anti-rabbit IgG (Sigma-Aldrich) was used as secondary antibody. Bands were visualised by enhanced chemiluminescence (Curix 60, AGFA Healthcare, Bonn, Germany) on X-ray films.

### Statistical analysis

Results are expressed as mean ± SD. Student’s t-test was used to test for significant differences between the cell lines. *P* values below 0.05 were considered statistically significant.

## Supplementary Information

Supplementary Information.

Supplementary Video 1.

Supplementary Video 2.

Supplementary Video 3.
