# Supplementary material for: Differences of the tumour cell glycocalyx affect binding of capsaicin-loaded chitosan nanocapsules
Source: Sci Rep. 2020 Dec 31;10:22443. doi: 10.1038/s41598-020-79882-y (PMC7775450; doi:10.1038/s41598-020-79882-y)
Supplement: Supplementary file 1 — Supplementary Information. [file 41598_2020_79882_MOESM1_ESM.pdf]

Supplementary Information

Differences of the tumour cell glycocalyx affect binding of capsaicin-loaded chitosan nanocapsules

*Lydia von Palubitzki[1], Yuanyuan Wang[1], Stefan Hoffmann[2], Sabine Vidal-y-Sy[1], Bernd Zobiak[3], Antonio V. Failla[3], Petra Schmage[4], Axel John[5], Anayancy Osorio-Madrazo[6], Alexander T. Bauer[1], Stefan W. Schneider[1], Francisco M. Goycoolea[7] and Christian Gorzelanny[1]**

[1] Experimental Dermatology, Department of Dermatology and Venereology, University Medical Center Hamburg-Eppendorf, Research Campus, Martinistraße 52, 20246 Hamburg, Germany

[2] Institute of Plant Biology and Biotechnology (IBBP), University of Münster, Schlossplatz 7-8, 48143 Münster, Germany

[3] Microscopy Imaging Facility, University Medical Center Hamburg-Eppendorf, Research Campus, Martinistraße 52, 20246 Hamburg, Germany

[4] Clinic of Periodontology, Preventive and Operative Dentistry, Center of Dental and Oral Medicine, University Medical Center Hamburg-Eppendorf, Martinistraße 52, 20246 Hamburg, Germany

[5] Department of Urology, University Medical Center of Ulm, Albert-Einstein-Allee 23, 89081 Ulm

[6] Institute of Microsystems Engineering (IMTEK), Freiburg Materials Research Center (FMF), and Freiburg Center for Interactive Materials and Bioinspired Technologies (FIT), University of Freiburg, 79104 Freiburg, Germany

[7] School of Food Science and Nutrition, University of Leeds, Woodhouse Lane, Leeds LS2 9JT, United Kingdom

Corresponding author: Christian Gorzelanny
Experimental Dermatology, Department of Dermatology and Venereology,
University Medical Center Hamburg-Eppendorf,
Research Campus, Martinistraße 52, 20246 Hamburg, Germany
Tel.: +49 40 7410 58976
Fax: +49 40 7410 52655
Email: c.gorzelanny@uke.de

Supporting information

To estimate the length of the heparan sulphate (L_{HS}) chains exposed by the T24 cells, we determined by nanoparticle tracking the number of Chi-NCs bound to the cell surface at c^* (N_{P,c^*} ~ 26476 particles per cell) and when Chi-NC binding approaches saturation ($N_{P,max}$ ~ 197749 particles per cell). In addition, we derived the average density of proteoglycans (D_{PG}) at the cell surface from stimulated emission depletion microscopy images (**Figure 2b**). Moreover, we assume that at saturating Chi-NC concentrations, the number of bound Chi-NCs ($N_{P,max}$) is related to the surface area of the cell (A_{cell}) according to $A_{cell} = N_{P,max}\,\pi r^2$, where r is the radius of the Chi-NCs. Supposing that at a low ionic strength the surface potential of the Chi-NCs approaches the zeta potential (ζ) we calculated the charge (Q_{NC}) of the Chi-NCs using the Grahame's equation:

$$Q_{NC} \approx \sqrt{8\epsilon_0 \epsilon kTcN_A}\,\sinh\left(\frac{e\zeta}{2kT}\right) \quad \text{(S1)}$$

where ζ of Chi-NCs was +50 mV. N_A is the Avogadro constant and the concentration of monovalent ions (c) was 2 mM. The charge on the T24 cells (Q_{cell}) was estimated as follows:

$$Q_{cell} = (N_{P,max} - N_{P,c^*})\;Q_{NC}\pi r^2 \quad \text{(S2)}$$

By combining Equations 1, S1 and S2, we derived the average number of monosaccharide units exposed by each cell (u_{cell}):

$$u_{cell} = \frac{Q_{cell} 4\xi^* \pi \epsilon_0 \epsilon kT}{e^2 A_{cell}} \quad \text{(S3)}$$

where ξ^* corresponds to ξ at c^* and was set to 1. Finally, the length of individual heparan sulphate chains (L_{HS}) was calculated as follows:

$$L_{HS} = \frac{u_{cell} l_u}{D_{PG} n_{HS}} f \quad \text{(S4)}$$

where the length of the monomeric unit (l_u) was 0.5 nm, D_{PG} was assumed to be 20 proteoglycan molecules per µm^2 and the average number of heparan sulphate chains per proteoglycan (n_{HS}) was assumed to be 4. Notably, we did not distinguish between heparan

sulphate and other related carbohydrates such as chondroitin sulphate and we assumed a 1:1 charge stoichiometry of the chitosan – heparan sulphate interaction, independent of highly or less sulphated heparan sulphate domains[1,2]. Because our flow cytometry experiments suggested that 16.7% of the Chi-NC binding was independent of heparan sulphate (**Figure 2d**) we corrected L_{HS} by the factor $f = 0.833$.

1 Boddohi, S., Moore, N., Johnson, P. A. & Kipper, M. J. Polysaccharide-based polyelectrolyte complex nanoparticles from chitosan, heparin, and hyaluronan. *Biomacromolecules* **10**, 1402-1409, doi:10.1021/bm801513e (2009).

2 Arguelles-Monal, W., Cabrera, G., Peniche, C. & Rinaudo, M. Conductimetric study of the interpolyelectrolyte reaction between chitosan and polygalacturonic acid. *Polymer* **41**, 2373-2378, doi:10.1016/s0032-3861(99)00396-1 (2000).

Supplemental Methods

qRT-PCR and PCR: Total RNA was extracted using the RNeasy Plus Mini Kit (Qiagen, Hilden, Germany) according to the manufacturer's protocol and qRT-PCR was performed using the Reverse Transcription System and the GoTaq qPCR Master Mix (Promega, Heidelberg, Germany). The following primer pairs were used: EXT1, 5′-GAGACAATGATGGGACAGACTTC-3′ and 5′-CTCTGTCGCTGGGCAAAG-3′; EXT2, 5′-CTGGGACCATGAGATGAATA-3′ and 5′-GATATCCCCAGGCATTTTGTA-3′; HPSE, 5′-ATGCTCAGTTGCTCCTGGAC-3′ and 5′-CTCCTAACTGCGACCCATTG-3′; SDC1, 5′-CTCAGGTGCAGGTGCTTTG-3′ and 5′-CTGCGTGTCCTTCCAAGTG-3′; SDC2, 5′-GATGACGATGACTACGCTTCTG-3′ and 5′-TGGAAGTGGTCGAGATGTTG-3′; SDC3, 5′-CTCCTTTCCCGATGATGAAC-3′ and 5′-CGACTCCTGCTCGAAGTAGC-3′; SDC4, 5′-GGCAGGAATCTGATGACTTTG-3′ and 5′-TCTAGAGGCACCAAGGGATG-3′; HAS1, 5′-TGCTCATCCTGGGCCTCA T-3′ and 5′-AATCTCCGAGCGCCTTGAA-3′; HAS2, 5′-AGTTGCCCTTTGCATCGCT-3′ and 5′-AGACTGACAGGCCCTTTCT-3′; HAS3, 5′-ACCAGTTCATCCACACGG-3′ and 5′-ACCTGGATGTAGTCCACCGA-3′; HYAL1, 5′-ATAGCTCCCAGCTGGGCA-3′ and 5′-AGATTGGGGTCACCAGCA-3′; HYAL2, 5′-TTGATGTGCAGGCCTCACCTA-3′ and 5′-CTCCTTAATGTCACGCACGAT-3′. Expression levels were normalised to the endogenous β-actin gene (5′-CATGTACGTTGCTATCCAGGC-3′ and 5′-CTCCTTAATGTCACGCACGAT-3′).

WGA staining: Sterilised cover slips were coated with 0.5% gelatin in a 24-well plate. T24 and UROtsa cells were seeded into the wells in RPMI-1640 medium supplemented as described above and allowed to attach overnight. The medium was removed, and encapsulated capsaicin was added at a concentration of 250 µM = 2.8×10^{12} particles mL^{-1} in RPMI-1640 medium without FCS. After incubation for 24 h, the medium was removed and the cells were fixed with 4% paraformaldehyde (Electron Microscopy Sciences, Hatfield,

USA), washed three times with PBS and blocked with 2% BSA in PBS for 1 h. We stained the cells overnight at 4 °C with Texas red-conjugated WGA (Thermo Fisher Scientific) diluted 1:1,000, and for 10 min with 2-(4-amidinophenyl)-6-indolecarbamidine dihydrochloride diluted 1:1,000 (Sigma-Aldrich). The cover slips were washed three times with incubation buffer and twice with distilled water. Mounted samples were analysed by fluorescence microscopy using an Observer z1 inverted microscope (Zeiss). Images were analysed with ImageJ v1.52.

Flow cytometry with salt-free HEPES-sucrose buffer: T24 cells were collected, centrifuged, and resuspended in isotonic salt-free 30 mM HEPES buffer containing 300 mM sucrose (pH 7.4). Chi-NCs were added at concentrations of 1.12×10^{11}, 2.24×10^{11}, 4.48×10^{11}, 8.96×10^{11}, 1.78×10^{12} particles mL^{-1} and the mixtures were incubated for 15 min on ice. . The cells were collected and centrifuged as above, although at higher Chi-NC concentrations the acceleration was increased to 1000×g. Binding activity was evaluated in triplicate as described above. The data were normalized to the PBS control and were presented as mean ± SD.

WST-1 assay: T24 and UROtsa cells (20,000 per well) were seeded into a 96-well plate and cultivated for 24 h in RPMI-1640 medium with 10% FCS at 37 °C in a humid, 5% CO_2 atmosphere. Prior to the treatment with Chi-NCs, cells were kept for 24 h in RPMI-1640 medium without FCS. Cells remained untreated (control) or were treated with 125 and 250 µM encapsulated capsaicin or equivalent amounts of empty Chi-NCs (1.4×10^{12}, 2.8×10^{12} particles mL^{-1}). Cells incubated with the cell disruptive 1% v/v Triton-X 100 (Sigma-Aldrich) were used as reference. WST-1 assay was conducted as proposed by the manufacturer (Sigma-Aldrich). Light absorbance (optical density) was measured at a wavelength of 450 nm by a multiplate reader (BioTek, PowerWave).

Supplemental data

Table S1: Expression of genes involved in heparan sulphate and hyaluronic acid biosynthesis. Shown are normalised gene expression levels (= $2^{-\Delta ct}$); β actin was selected as housekeeping gene.

gene	T24	UROtsa	MV3	BLM
	mean±SD*	mean±SD*	mean±SD*	mean±SD*
EXT1	$9\times10^{-3}\pm4\times10^{-3}$	$8\times10^{-3}\pm2\times10^{-3}$	$2\times10^{-2}\pm1\times10^{-3}$	$2\times10^{-2}\pm7\times10^{-3}$
EXT2	$2\times10^{-2}\pm5\times10^{-3}$	$1\times10^{-2}\pm3\times10^{-3}$	$2\times10^{-2}\pm4\times10^{-3}$	$4\times10^{-2}\pm2\times10^{-2}$
HPSE	$2\times10^{-4}\pm3\times10^{-5}$	$2\times10^{-3}\pm3\times10^{-4}$	$6\times10^{-4}\pm4\times10^{-5}$	$1\times10^{-3}\pm4\times10^{-4}$
SDC1	$1\times10^{-2}\pm2\times10^{-3}$	$5\times10^{-2}\pm4\times10^{-3}$	$2\times10^{-2}\pm5\times10^{-3}$	$1\times10^{-2}\pm3\times10^{-3}$
SDC2	n.d.	n.d.	$2\times10^{-4}\pm3\times10^{-5}$	n.d.
SDC3	$1\times10^{-3}\pm5\times10^{-4}$	$2\times10^{-4}\pm7\times10^{-5}$	$8\times10^{-4}\pm3\times10^{-4}$	$7\times10^{-4}\pm2\times10^{-4}$
SDC4	$4\times10^{-2}\pm2\times10^{-2}$	$5\times10^{-2}\pm7\times10^{-3}$	$2\times10^{-2}\pm9\times10^{-4}$	$3\times10^{-2}\pm2\times10^{-2}$
HAS1	n.d.	n.d.	n.d.	n.d.
HAS2	$1\times10^{-4}\pm1\times10^{-4}$	n.d.	$8\times10^{-4}\pm4\times10^{-4}$	$6\times10^{-4}\pm4\times10^{-4}$
HAS3	n.d.	$9\times10^{-5}\pm1\times10^{-4}$	n.d.	n.d.
HYAL1	n.d.	n.d.	n.d.	n.d.
HYAL2	$6\times10^{-5}\pm8\times10^{-5}$	n.d.	$1\times10^{-4}\pm2\times10^{-4}$	$8\times10^{-5}\pm1\times10^{-5}$

n.d. = not detectable
*n = 3

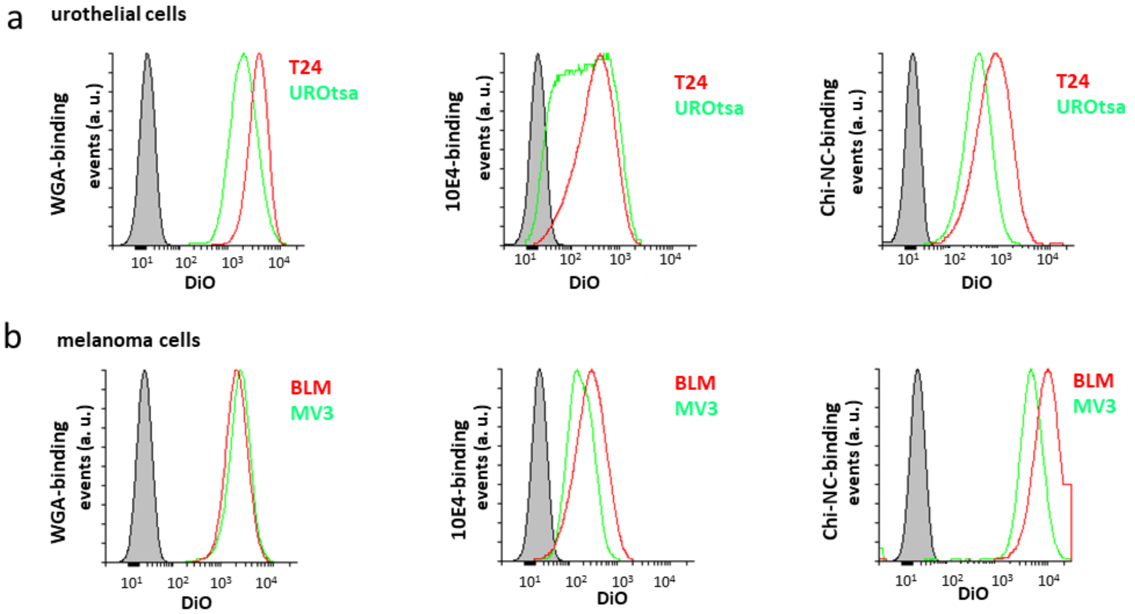

Figure S1: Representative flow cytometry data supporting Figure 3. (**a-b**) Comparison of the WGA-binding ability, heparan sulphate expression (10E4-binding) and the capacity to interact with Chi-NCs (Chi-NC-binding) in urothelial cells (a) and melanoma cells (b). Chi-NC concentration = 1.78×10^{12} particles mL^{-1}. The grey curve shows the autofluorescence of untreated cells.

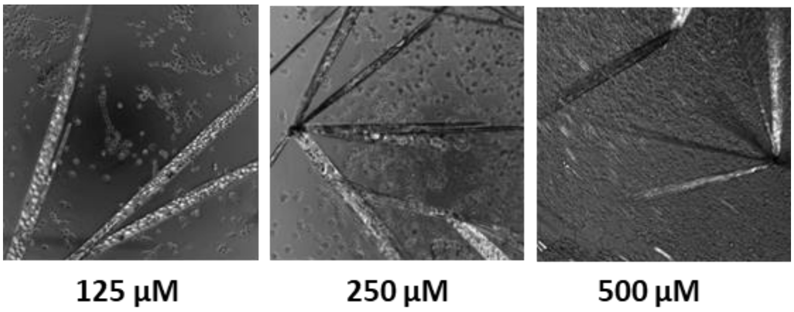

Figure S2: Precipitation of free capsaicin and formation of insoluble crystals in the culture medium after 22 h. Capsaicin ≥95% was dissolved in ethanol at a concentration of 24 mg mL^{-1}. In the culture medium was a concentration of 125 µM, 250 µM or 500 µM free capsaicin.

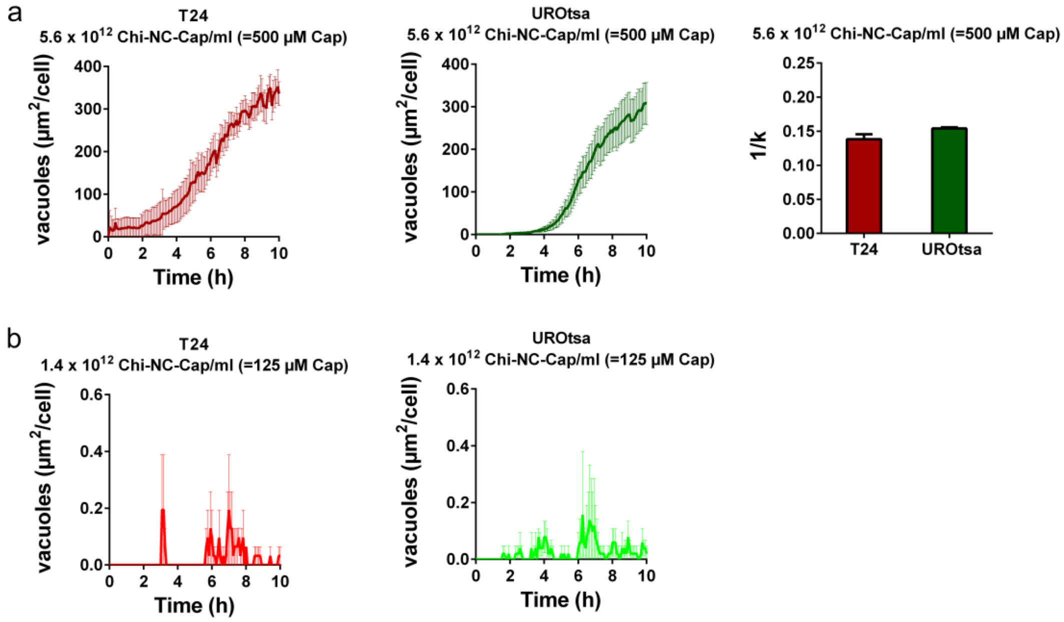

Figure S3: Vacuole formation at 500 µM and 125 µM encapsulated capsaicin. (**a**) Analysis of fluorescence microscopy results after treatment of T24 and UROtsa cells with high concentrations Chi-NC-Cap (5.6×10^{12} particles mL^{-1} = 500 µM capsaicin). Bar diagram shows the ability of the cells to deposit capsaicin in vacuoles (k^{-1}). Data are presented as mean ± SD (n = 3). (**b**) Analysis of fluorescence microscopy results after treatment of T24 and UROtsa cells with low concentrations of Chi-NC-Cap (1.4×10^{12} particles mL^{-1} = 125 µM capsaicin).

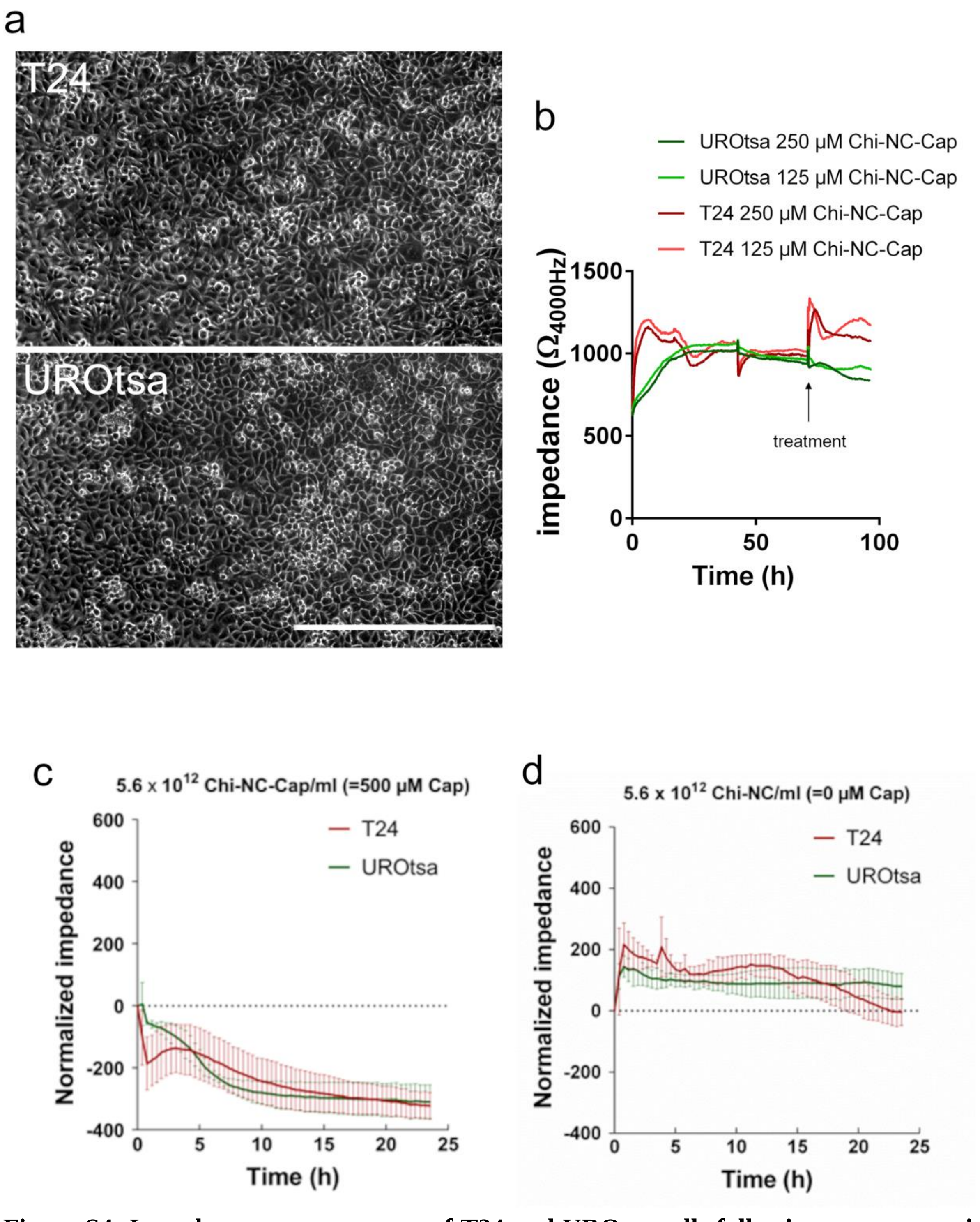

Figure S4: Impedance measurements of T24 and UROtsa cells following treatment with capsaicin-loaded or empty Chi-NCs. (a) Representative brightfield microcopic image of T24 and UROtsa cells. Both cell lines form a confluent cell layer after 74 h of cultivation. Because gold electrodes of the ECIS slide prevent proper brightfield microscopy, images were taken from an equivalent slide without gold electrodes but with the same growth area, same geometry and same surface coating. Scale bar corresponds to 500 µm. (b) Representative ECIS measurement showing the absolute impedance measured at 4000 Hz. The measurement was started immediately after cell seeding. Culture medium was exchanged every day. After 72 h of cultivation, cells were treated with Chi-NCs. (c) Treatment with Chi-

NC-Cap (NCs (5.6×10^{12} particles mL^{-1} = 500 µm capsaicin) showing the dose-dependent capsaicin-induced change in cell layer impedance Z. (d) Control experiments with empty Chi-NCs (no capsaicin) at equivalent nanoparticle concentrations (5.6×10^{12} particles mL^{-1}). Data are presented as mean ± SD (n = 4).

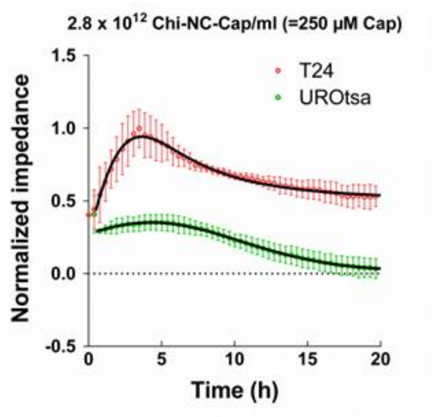

Figure S5: **Curve fit (black solid line) of ECIS results (250 µM encapsulated capsaicin) using Equation 2**. Results shown in Figure 5c-f were further derived from these fitted data.

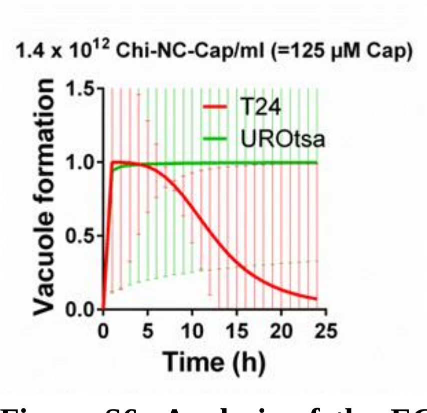

Figure S6: Analysis of the ECIS data. Vacuole formation as a function of time at low concentrations of Chi-NC-Cap (1.4×10^{12} particles mL^{-1} = 125 µM capsaicin) was not analysable. Data are presented as mean ± SD (n = 3).

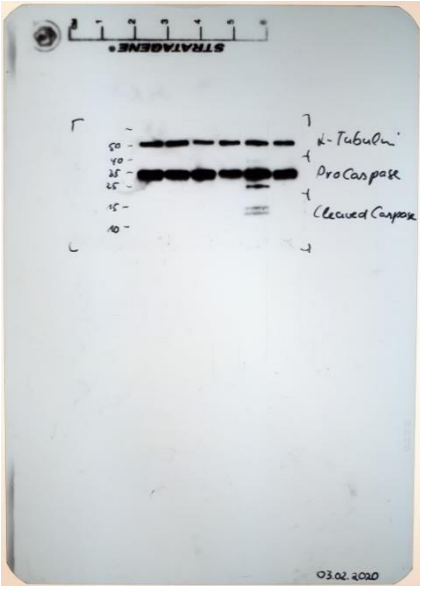

lane 1 = T24 control
lane 2 = UROtsa control
lane 3 = T24 125 µM capsaicin
lane 4 = UROtsa 125 µM capsaicin
lane 5 = T24 250 µM capsaicin
lane 6 = UROtsa 250 µM capsaicin

Figure S7: Unprocessed western blot image related to Figure 5h. Detection of procaspase-3, cleaved caspase-3 and α-tubulin by western blot in lysates of T24 and UROtsa cells treated with Chi-NC-Cap (1.4×10^{12} particles mL^{-1} = 125 µM capsaicin and 2.8×10^{12} particles mL^{-1} = 250 µM capsaicin).

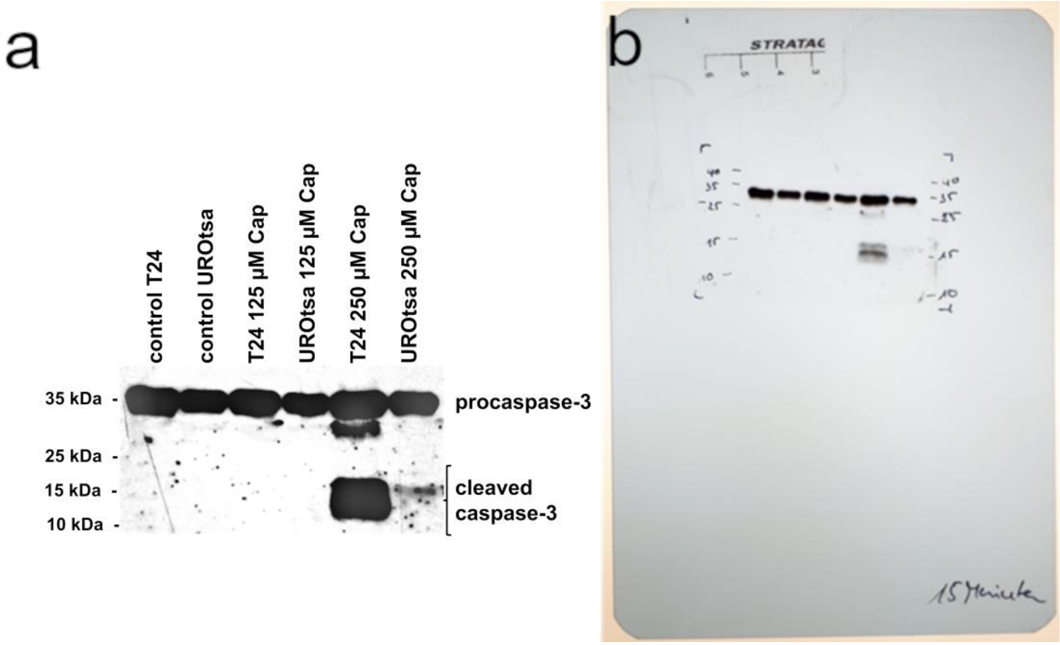

Figure S8: Enhanced western blot of caspase 3. (**a**) To detect weak signals of cleaved caspase-3, western blot membrane was exposed for 15 minutes to X-ray films. Very low levels of cleaved caspase 3 were detectable in UROtsa cells treated with 250 µM encapsulated capsaicin. (**b**) Unprocessed western blot image related to Figure S8a.

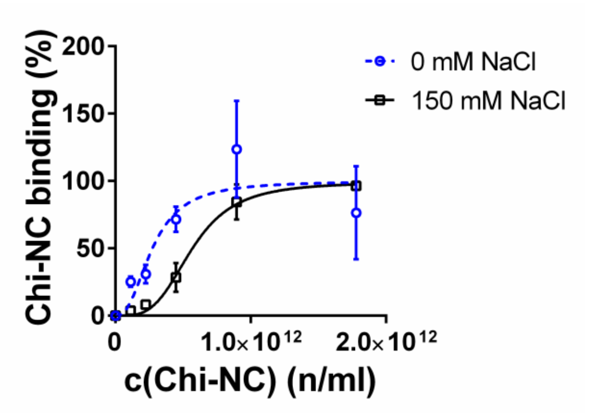

Figure S9: Impact of the ionic strength on Chi-NC binding. Chi-NC binding to T24 cell under nearly salt-free conditions (0 mM NaCl) compared to physiological salt conditions (150 mM NaCl). Chi-NC binding was measured by flow cytometry. At low Chi-NC concentrations, T24 cells, which were suspended in 0 mM NaCl, bound more Chi-NCs than T24 cells, which were suspended in 150 mM NaCl. This suggests that at nearly salt free conditions the proposed counterion condensation was abolished. High SD at high Chi-NC concentrations was most likely related to cellular disintegration, due to the strongly increased ability of the cells to interact with the Chi-NCs. Data are presented as mean ± SD (n = 3).

Video S1: Representative video taken during live cell fluorescence microscopy experiments of T24 cells.

The cells were treated with 250 µM capsaicin corresponding to 2.8×10^{12} particles mL^{-1}. Scale bars = 50 µm. Time = hh:mm:ss.

Video S2: Representative video taken during live cell fluorescence microscopy experiments of UROtsa cells.

The cells were treated with 250 µM capsaicin corresponding to 2.8×10^{12} particles mL^{-1}. Scale bars = 50 µm. Time = hh:mm:ss.

Video S3: Representative video taken during live fluorescence microscopy confirms that Chi-NCs rapidly bind to the cell surface.

Chi-NC binding (5.6×10^{12} particles mL^{-1}) was also associated with a slight cellular detachment, which may coincide with reduced cellular motility. Scale bars = 50 µm. Time = mm:ss.
